# Combined Transcriptome and Proteome Analysis of Immortalized Human Keratinocytes Expressing Human Papillomavirus 16 (HPV16) Oncogenes Reveals Novel Key Factors and Networks in HPV-Induced Carcinogenesis

**DOI:** 10.1128/mSphere.00129-19

**Published:** 2019-03-27

**Authors:** Ruwen Yang, Jana Klimentová, Elke Göckel-Krzikalla, Regina Ly, Nadine Gmelin, Agnes Hotz-Wagenblatt, Helena Řehulková, Jiří Stulík, Frank Rösl, Martina Niebler

**Affiliations:** a Division of Viral Transformation Mechanisms, German Cancer Research Center (DKFZ), Heidelberg, Germany; b Department of Molecular Pathology and Biology, Faculty of Military Health Sciences, University of Defense, Hradec Králové, Czech Republic; c Core Facility Omics IT and Data Management (ODCF), German Cancer Research Center (DKFZ), Heidelberg, Germany; Northwestern University

**Keywords:** HPV, RNA-Seq, SILAC, TCGA, cervical cancer, head and neck cancer, integrated analysis

## Abstract

Although the role of high-risk human papillomaviruses (hrHPVs) as etiological agents in cancer development has been intensively studied during the last decades, there is still the necessity of understanding the impact of the HPV *E6* and *E7* oncogenes on host cells, ultimately leading to malignant transformation. Here, we used newly established immortalized human keratinocytes with a well-defined HPV16 *E6E7* expression cassette to get a more complete and less biased overview of global changes induced by HPV16 by employing transcriptome sequencing (RNA-Seq) and stable isotope labeling by amino acids in cell culture (SILAC). This is the first study combining transcriptome and proteome data to characterize the impact of HPV oncogenes in human keratinocytes in comparison with their virus-negative counterparts. To enhance the informative value and accuracy of the RNA-Seq data, four different bioinformatic workflows were used. We identified potential novel upstream regulators (e.g., CNOT7, SPDEF, MITF, and PAX5) controlling distinct clusters of genes within the HPV-host cell network as well as distinct factors (e.g., CPPED1, LCP1, and TAGLN) with essential functions in cancer. Validated results in this study were compared to data sets from The Cancer Genome Atlas (TCGA), demonstrating that several identified factors were also differentially expressed in cervical squamous cell carcinoma and endocervical adenocarcinoma (CESC) and HPV-positive head and neck squamous cell carcinomas (HNSCs). This highly integrative approach allows the identification of novel HPV-induced cellular changes that are also reflected in cancer patients, providing a promising omics data set for future studies in both basic and translational research.

**IMPORTANCE** Human papillomavirus (HPV)-associated cancers still remain a big health problem, especially in developing countries, despite the availability of prophylactic vaccines. Although HPV oncogenes have been intensively investigated for decades, a study applying recent advances in RNA-Seq and quantitative proteomic approaches to a precancerous model system with well-defined HPV oncogene expression alongside HPV-negative parental cells has been missing until now. Here, combined omics analyses reveal global changes caused by the viral oncogenes in a less biased way and allow the identification of novel factors and key cellular networks potentially promoting malignant transformation. In addition, this system also provides a basis for mechanistic research on novel key factors regulated by HPV oncogenes, especially those that are confirmed *in vivo* in cervical cancer as well as in head and neck cancer patient samples from TCGA data sets.

## Abstract (precis)

Human papillomavirus (HPV)-associated cancers still remain a big health problem, especially in developing countries, despite the availability of prophylactic vaccines. Although HPV oncogenes have been intensively investigated for decades, a study applying recent advances in RNA-Seq and quantitative proteomic approaches to a precancerous model system with well-defined HPV oncogene expression alongside HPV-negative parental cells has been missing until now. Here, combined omics analyses reveal global changes caused by the viral oncogenes in a less biased way and allow the identification of novel factors and key cellular networks potentially promoting malignant transformation. In addition, this system also provides a basis for mechanistic research on novel key factors regulated by HPV oncogenes, especially those that are confirmed *in vivo* in cervical cancer as well as in head and neck cancer patient samples from TCGA data sets.

## INTRODUCTION

High-risk human papillomaviruses (hrHPVs) are not only the etiological agents for cervical cancer (1, 2), but also associated with other malignancies, including head and neck (60%) (3), anal (93%) (4), vulvar (69%), vaginal (75%), and penile cancers (47%) (5). Among all hrHPV types, HPV16 is the most prevalent one, posing a heavy health burden on both males and females (5). Since 2006, three prophylactic HPV vaccines have been widely used worldwide to provide protection against hrHPV-related cancers (6, 7). However, these cancers still remain a challenge in countries where screening and vaccination are unavailable (8). Recent global cancer statistics from GLOBOCAN show that cervical cancer is still a leading cause of death in 42 countries in Sub‐Saharan Africa and Southeast Asia (9). Meanwhile, therapeutic vaccines targeting existing persistent infections or hrHPV-positive tumors are still in their infancy (10). Further studies to identify novel factors and pathways regulated by hrHPV are mandatory to provide new insights into virus-host interactions to allow more-effective diagnoses and treatments of hrHPV-associated cancers.

Taking an evolutionary viewpoint into account (11), HPVs are well-selected entities whose oncoproteins always attack central hubs within key regulatory pathways to gain a selective advantage (12). The hrHPV E6 and E7 oncoproteins target p53 and pRB, two of the key cellular tumor suppressors, for proteasomal degradation (13, 14), and their ectopic expression is sufficient to immortalize primary keratinocytes (15, 16). Besides sustaining proliferative signaling, promoting genome instability, and resisting cell death, hrHPV E6 and E7 also account for immune escape, angiogenesis, and the formation of a proproliferative microenvironment, making them key players in tumor development by affecting the hallmarks of cancer (17, 18).

Interactions between HPV oncogenes and host cells have been intensively investigated during the past 15 years, during which time methods such as microarray analysis, transcriptome sequencing (RNA-Seq), or proteomics revealed global changes caused by E6 and E7. In principle, there are different ways to investigate the role of HPV oncogenes in host cell transformation. The most common approaches were either to express or to knock down *E6* and/or *E7* to study the changes in host transcriptome profiling (19–23). In addition, available cell lines or clinical samples with different progression states were used to examine the role of either distinct HPV oncogenes or the whole viral genome in an episomal or integrated state (22, 24, 25). Moreover, there were also attempts to understand the host transcriptome of cells where a productive cycle of HPV was taking place (26). The generation of new HPV progenies can occur only when the host cell is able to differentiate (27). In contrast, HPV-induced cancer is considered to be an evolutionary accident with a dead end for the viral life cycle (28).

In the present study, we used immortalized human keratinocytes (normal oral keratinocytes [NOKs]) (29) with a well-defined expression of HPV16 oncogenes and the corresponding parental negative-control cells. We intentionally chose immortalized cells over primary cells, allowing a certain number of cell doublings to guarantee sufficient amino acid incorporation for stable isotope labeling by amino acids in cell culture (SILAC). Furthermore, the use of immortalized cells also permits an unlimited number of follow-up experiments in the HPV-negative parental cell line without bias being created by differentiation of primary cells or by variations among different donors (30, 31). Using this *in vitro* model, transcriptome and quantitative proteome data were combined for the first time to identify novel regulators that putatively contribute to host cell transformation. Moreover, to enhance the validity of RNA-Seq analysis, results were processed by different bioinformatic methods to get a more complete and less biased overview of global changes caused by HPV16 oncogenes. Key findings were supported by data from various cancer entities in The Cancer Genome Atlas (TCGA).

## RESULTS

### Expression of HPV16 *E6* and *E7* in normal keratinocytes leads to degradation of p53 and pRb.

**FIG 1:**
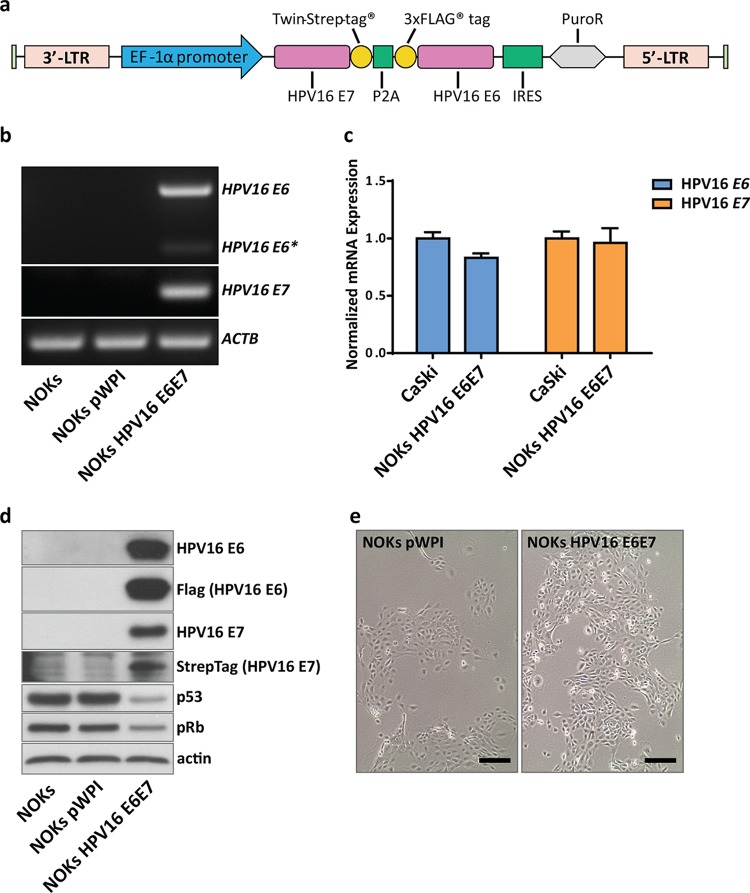
Establishment of a keratinocyte cell line expressing HPV16 *E6* and *E7*. (a) Schematic representation of the vector construct used to stably express *E6* and *E7* in NOKs. Strep-tagged *E7* and Flag-tagged *E6*, separated by a P2A sequence, were cloned into the pWPI vector for lentiviral packaging. NOK cells were subsequently transduced and selected with puromycin. Pools of oncogene-transduced or empty vector control cells were used for all further studies. 3′-LTR, 3′ long terminal repeat; IRES, internal ribosome entry site. (b) Semiquantitative PCR of transduced NOKs. RNA of untransduced (NOKs), NOKs with an empty vector control (NOKs pWPI), and NOKs with oncogene-transduced cells (NOKs HPV16 E6E7) was reverse transcribed, and PCR was performed with primers for HPV16 E6/E6* and *E7*. *ACTB* served as an internal control. (c) Quantitative PCR comparing relative transcript levels of *E6* and *E7* from NOKs HPV16 E6E7 with the HPV16-positive cancer cell line CaSki. Transcript levels were normalized to those of *TOP1* as a housekeeping gene. (d) Western blot analyses of untransduced and transduced NOKs. Protein lysates (30 µg) were analyzed for the presence of HPV16 E6 and E7 using oncoprotein-specific antibodies as well as anti-Flag (E6) antibody or streptavidin-HRP (E7). Levels of p53 and pRb were analyzed to confirm the functionality of the viral proteins. Actin served as a loading control. (e) Light microscopic image of transduced cells prior to RNA extraction. Scale bar = 200 µM.

Immortalized female human oral keratinocytes stably expressing the HPV16 oncogenes *E6* and *E7* after lentiviral transduction as well as their HPV-negative counterparts generated with an empty vector were used for transcriptome and proteome analyses. During a normal infection cycle as well as when integrated into the host genome, *E6* and *E7* are transcribed into a polycistronic mRNA, and the amounts of oncoproteins depend on alternative splicing occurring within the open reading frame (ORF) of *E6* (32). These as yet not fully understood splicing events usually result in either higher levels of full-length E6, leading to decreased translation of E7, or shortened variants of E6 (E6*), allowing more-efficient translation of E7 (33). To circumvent unpredictable viral splicing events that can be affected by different stimuli (34), the “self-cleaving” peptide sequence P2A (35) was placed between the *E6* and *E7* ORFs. For purposes of detection and further investigation, Twin-Strep-tag (*E7*) and a 3×FLAG tag (*E6*) were added (Fig. 1a). The cassette was placed under the control of the EF1α promoter to ensure constitutive expression. Corresponding control cells, transduced with an empty lentiviral vector, were generated in parallel (NOKs pWPI). To avoid integration site-dependent effects (36), pools of transduced cells at early passage numbers (within 10 passages after puromycin selection) were used for all further experiments.

As shown in Fig. 1b, expression of both oncogenes and only minor splicing events of the *E6* gene were observed. In order to evaluate whether *E6* and *E7* mRNA levels in our *in vitro* model (NOKs HPV16 E6E7) are comparable to those of a patient-derived cell line containing integrated HPV16, we used the HPV16-positive cancer cell line CaSki for comparison. Quantitative PCR (qPCR) analysis showed that the amounts of viral transcripts are similar in both cell lines (Fig. 1c). Production of the corresponding oncoproteins was verified by Western blot analyses using antibodies against the genuine proteins or their respective tags (Fig. 1d). The functionality of the tagged viral oncoproteins was indirectly verified by decreased levels of p53 (targeted by E6) and pRb (targeted by E7). In addition, both NOKs pWPI and NOKs HPV16 E6E7 maintained their epithelial morphology in culture (Fig. 1e).

### Changes in the transcriptome and proteome of NOKs HPV16 E6E7 compared to those of NOKs pWPI.

**FIG 2:**
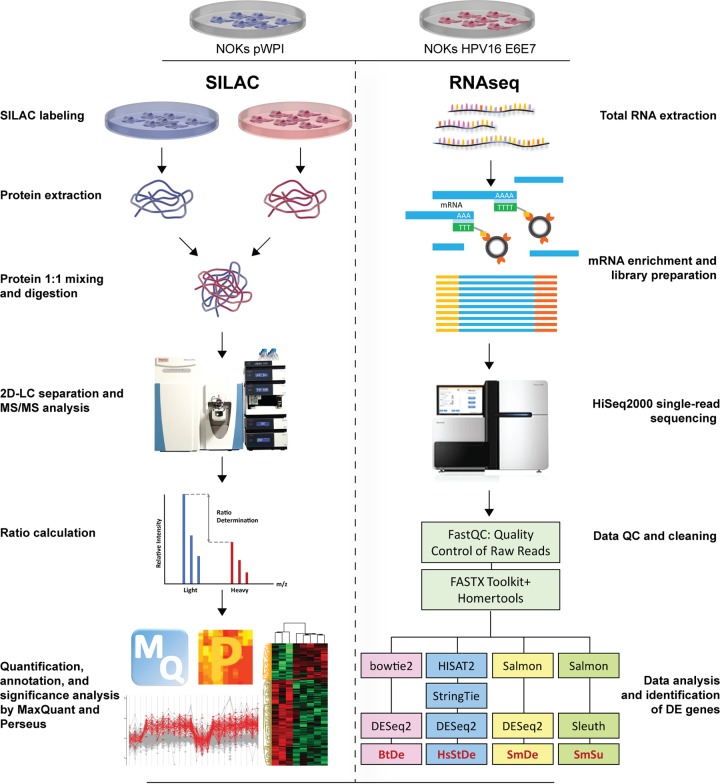
Schematic overview of the experimental procedures used in this study. Protein and RNA were extracted from NOKs pWPI and NOKs HPV16 E6E7 to perform SILAC and RNA-Seq experiments in parallel. Proteins from the two cell lines were quantified and mixed at a ratio of 1:1, followed by trypsin digestion, two-dimensional LC separation of peptides, and MS/MS analysis. Protein identification, abundance calculation, and annotation were performed with MaxQuant. Perseus was used for comparison and statistical analysis. mRNA was enriched for library preparation and single-read sequencing. Raw reads were put through the FASTX Toolkit and Homertools to remove adaptor sequences and reads with bad quality. Four different sets of tools, including Bowtie2 plus DESeq2, HISAT2 plus StringTie plus DESeq2, Salmon plus DESeq2, and Salmon plus Sleuth were used for read alignment and gene expression quantification. The outputs of each set of tools were abbreviated BtDe, HsStDe, SmDe, and SmSu, respectively. Data from SILAC and RNA-Seq were combined for pathway analysis and interpretation of biological functions. Illustrations for the dish, cell, and protein were obtained from Library of Science and Medical Illustrations (http://www.somersault1824.com) and are used here with permission.

**FIG 3:**
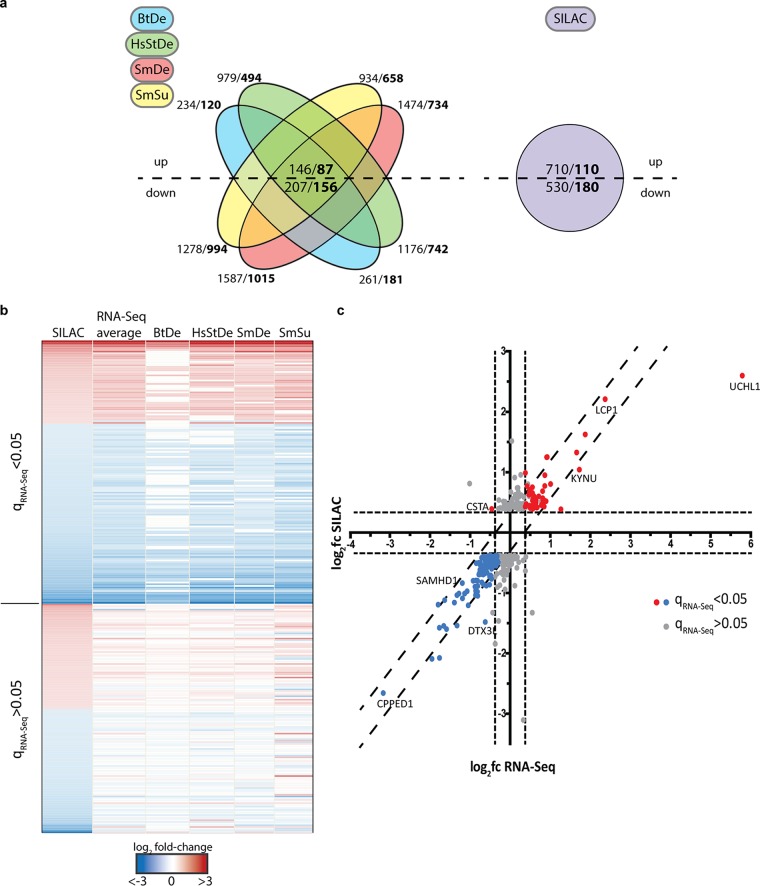
Graphic representation of differentially regulated proteins obtained by SILAC and RNA-Seq analyses. (a) Diagrams show the numbers of DE genes in RNA-Seq (left) and SILAC (right) results. Up- and downregulated factors were used separately for plotting. Numbers represent the factors with *q* values of <0.05 (RNA-Seq) or *P* values of <0.05 (SILAC). In bold, the numbers of factors with fold changes of at least ±1.5 (RNA-Seq) or ±1.3 (SILAC) are shown. For RNA-Seq results, numbers of DE genes shared by all four methods are shown in the center. (b) Heatmap of the 290 significantly altered proteins with at least a ±1.3 fold change from the corresponding genes in the RNA-Seq analysis using four bioinformatic workflows. One hundred fifty-five genes (upper part of heat map) displayed significant changes (*q* values of <0.05) in at least two of the four RNA-Seq data sets, whereas the other genes showed significant alterations in only one or none of the lists. (c) Graphic representation of the correlation of log_2_ fold changes observed for factors from panel b between SILAC and RNA-Seq results. Values for RNA-Seq represent the averaged log_2_ fold changes from all four lists. Blue and red dots show factors with *q* values of <0.05 in the RNA-Seq analysis that were significantly changed from values from the SILAC analysis. Gray dots show genes with *q* values of >0.05. Dashed lines represent cutoffs of ±1.5 fold change (RNA-Seq, *x* axis) or ±1.3 fold change (SILAC, *y* axis).

In order to assess the impact of oncogene expression on host cells, NOKs pWPI and NOKs HPV16 E6E7 were used for RNA-Seq and SILAC experiments according to the workflows outlined in Fig. 2. For the SILAC approach, among a total of 3,670 detected proteins, 290 were considered differentially expressed (DE), with 110 up- and 180 downregulated, using a cutoff *t* test *P* of <0.05 and a fold change bigger than 1.3 or smaller than 0.7 (Fig. 3a). Due to the heterogeneity in the process of different workflows for RNA-Seq data analysis (37, 38), four different combinations of tools were chosen to get a less biased view of DE genes. As shown in the Venn diagram in Fig. 3a, when the same cutoff (*q *<* *0.05) was applied to the results from all methods (with fold changes of at least ±0.5), the combination of Bowtie2 and DESeq2 (BtDe) generated the smallest number of DE genes (301 in total; 120 up- and 181 downregulated). Meanwhile, the combination of Salmon and DESeq2 (SmDe) produced the maximum number of DE genes (1,749 in total; 734 up- and 1,015 downregulated). Eighty-seven upregulated and 156 downregulated genes were finally found in results from all four methods.

To analyze whether the changes identified by SILAC and RNA-Seq correlate, the results of the proteome study were compared to the results from all four sets of the transcriptome data. Here, 155 genes whose corresponding proteins were significantly deregulated showed similar changes in at least two RNA-Seq DE gene lists, with *q *of <0.05 (Fig. 3b). The remaining factors showed significant changes in only one or none of the RNA-Seq lists. When the log_2_ fold changes at the protein level were plotted against the average log_2_ fold changes throughout the four RNA-Seq lists, 154 out of 155 factors showed a correlation (Fig. 3c; see Table S3 in the supplemental material). Only CSTA was downregulated at the mRNA level but upregulated at the protein level.

Furthermore, initial analysis of RNA-Seq and SILAC data identified factors (Table S3) that were already shown to be affected by HPV in multiple previous studies, including the upregulated genes *IL1A* (39), *UCHL1* (40), *TYMS* (41), *EGFR* (42), *TP63* (43), and *PCNA* (44) and the downregulated genes *STAT1* (45), *FN1* (46), and *ISG15* (47). In other words, the validation of aforementioned findings served as an internal reference for the reliability of this cell system to identify new factors with important functions in HPV-related cancers. Genes that are highly modulated in all lists were validated by qPCR (Fig. S1a). Here, the expression of *CPPED1*, *OAS2*, *OAS3*, *FN1*, *SAMHD1*, and *ISG15* was significantly downregulated, while that of *KYNU*, *LCP1*, *UCHL1*, and GAGE12H was upregulated, comparable to the results from RNA-Seq. Significantly modulated proteins from SILAC results were validated by Western blotting (Fig. S1b).

10.1128/mSphere.00129-19.1 FIG S1 Validation of significantly altered genes and proteins. (a) qPCR analysis of selected DE genes. Bar charts are results from qPCR analysis using *TOP1* expression as an internal control. NOKs pWPI samples were set to 1 as a baseline value to which transcript levels of NOKs HPV16 E6E7 were normalized. Error bars refer to standard deviations for three independent experiments. Statistical analysis was done by unpaired *t* test with Welch’s correction. *, *P* < 0.05; **, *P* < 0.01; ***, *P* < 0.001; ****, *P* < 0.0001. (b) Western blot analysis of selected proteins whose levels were shown to be significantly altered in SILAC. β-Actin served as a loading control. Fifty micrograms of lysates was used for this experiment. Download FIG S1, PDF file, 0.8 MB. Copyright © 2019 Yang et al. 2019 Yang et al. This content is distributed under the terms of the Creative Commons Attribution 4.0 International license.

Next, we analyzed whether general transcriptome changes observed here are in accordance with those of other studies. For this purpose, we took the 155 factors identified above (Fig. 3b) and compared them to the results of three other studies performed with primary foreskin keratinocytes (48), primary lung fibroblasts (49), and primary cervical keratinocytes (50) (Table S4). Although certain discrepancies can be seen among all four studies, we found a large number of genes, such as *TAGLN*, *TGM2*, *FN1*, *AURKB*, and *SAMD9*, showing changes with the same tendencies, especially when this study was compared to the RNA-Seq-based study of primary foreskin keratinocytes (48).

### IPA analyses identified new upstream regulators putatively involved in HPV-driven carcinogenesis.

**FIG 4:**
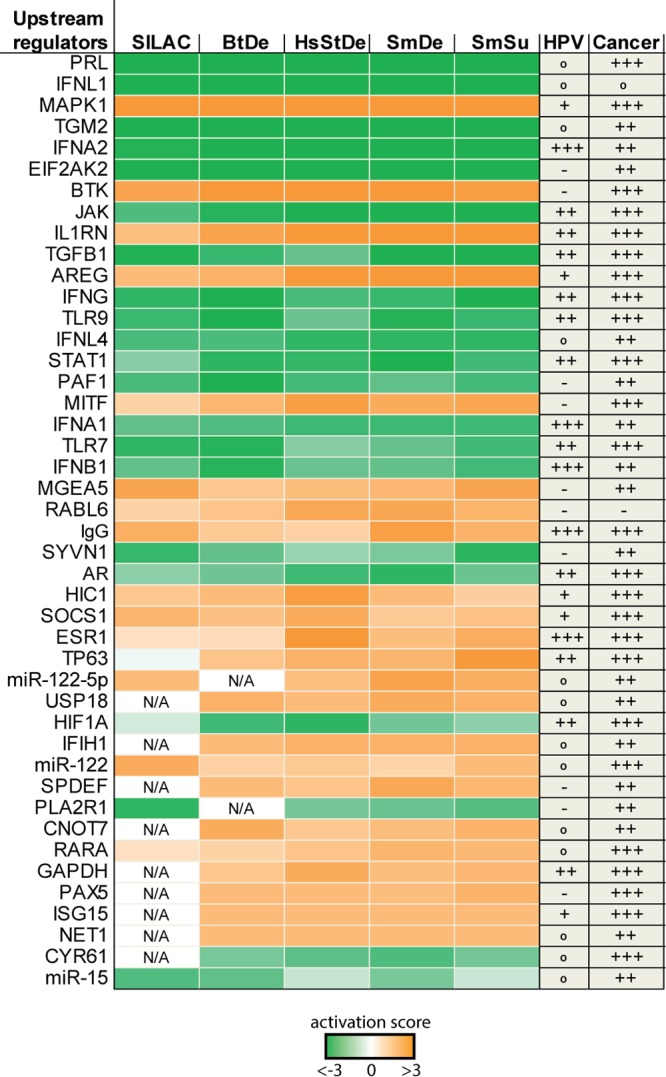
Activity profile of upstream regulators predicted by pathway analysis. Significantly deregulated factors obtained by different bioinformatic processing methods and SILAC data were individually analyzed using the core analysis function of Ingenuity Pathway Analysis (IPA) and subsequently compared with each other. Depicted are predicted upstream regulators displaying activation factors higher than 3 (orange) or lower than −3 (green). Pubmed citations with HPV or cancer in previous studies are indicated as +++ (mentioned >100 times), ++ (mentioned >10 times), + (mentioned at least 5 times), ° (mentioned <5 times), and – (not reported).

In order to determine whether changes in the transcriptome and proteome can be attributed to a small number of regulatory proteins, we subjected all five lists of significantly changed genes/proteins to comparative pathway analyses using Qiagen’s Ingenuity Pathway Analysis (IPA) platform. For this purpose, the results of individual core analyses and the data sets obtained from the four RNA-Seq lists as well as SILAC results were compared to each other. Figure 4 shows the 44 upstream regulators with the highest activity scores (activation Z-score), including regulatory factors that were previously reported to be affected by HPV, like interferons (51), *STAT1* (45), *TGFB1* (52), and the estrogen receptor (*ESR1*) (53). Some of the predicted upstream regulators were also supported by data from other studies (Table S6), such as *RABL6*, *CDKN1A*, *TNF*, and *EHF*.

Of the 3,837 factors whose levels were significantly altered in at least one of the five data sets, 15.27% (586 factors) are predicted to be regulated by at least one of the 44 identified upstream regulators (Table S5). The respective values of this ratio in the individual data sets ranged from 14.04% in SmDe (430 out of 3,063 genes) to up to 32.26% in BtDe (160 out of 496 genes). This highlights that oncogene-induced changes of a few upstream regulating factors are sufficient to cause major alterations both in the transcriptome and in the proteome.

**FIG 5:**
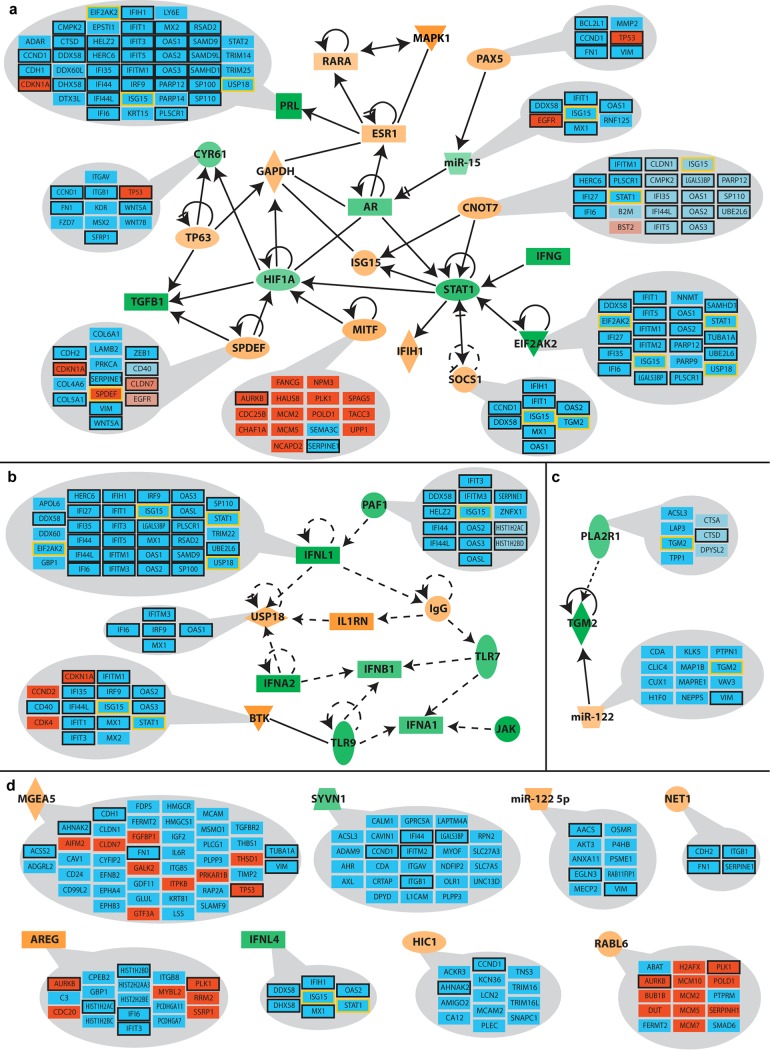
Reported association of predicted upstream regulators and their regulated genes. Putative upstream regulators were identified by IPA based on the observed up- or downregulation of the indicated genes (blue boxes, downregulation; red boxes, upregulation). Light-hued boxes indicate genes for which an association with the regulator was not yet fully confirmed. The average predicted activity changes of regulators are represented by the intensity of their respective color (orange, increased activity; green, decreased activity). Black-framed boxes depict a regulation of the respective gene by more than one of the identified upstream regulators. Yellow-framed genes are themselves predicted to be upstream regulators. Upstream regulators were grouped into clusters according to their previously reported associations. (a) Cluster of factors directly involved with each other (solid lines); (b and c) clusters of predicted factors indirectly associated with each other (dashed lines); (d) upstream regulators without reported links to any of the other predicted factors from panels a to c.

In order to assess the influence of these upstream regulators, we conducted further analyses, which revealed clusters that can be partially organized in a hierarchical structure (Fig. 5). The largest identified cluster consists of 21 regulators that are all directly connected to each other (Fig. 5a). Moreover, some of the upstream regulators are situated downstream of others. *STAT1* (a well-documented factor in gamma interferon signaling that is decreased upon HPV oncogene expression) not only displays a self-regulatory loop but additionally is regulated by five upstream factors, including a transcription factor (*CNOT7*) shown to negatively affect *STAT1* expression (54). Another central node is the hypoxia-inducible transcription factor (HIF1α). Apart from its regulation by *STAT1*, it is shown to be downstream of two other transcription factors, namely, *SPDEF* and *MITF*. Notably, knockdown of *SPDEF* was previously shown to increase the expression of HIF1α (55).

A second cluster links 12 upstream regulators by indirect associations (Fig. 5b). This cluster is comprised mainly of innate immunity-associated factors, like interferons and Toll-like receptors, whose activity is predicted to be predominantly downregulated based on the expression of their respective target genes. Apart from an upstream regulation of *TGM2* by *PLA2R1* and miR-122 (Fig. 5c), the remaining eight upstream regulators do not have any reported association with any of the other factors analyzed here (Fig. 5d). Their respective functions have previously been mentioned in different cancer backgrounds but not yet in the context of HPV (Fig. 4).

Since the observed HPV-induced proteome changes occurred mainly at the transcriptional level (Fig. 3), we focused on transcriptional regulators because their activity changes can affect multiple downstream targets. In addition to *STAT1*, *HIF1α*, and *TP63*, which have been reported to be affected by HPV before (43, 45), pathway analysis predicted *CNOT7*, *SPDEF*, *MITF*, *PAX5*, and *HIC1* as major novel regulators (Fig. 4 and 5). In general, these transcription factors have been shown to regulate immunity, proliferation, differentiation, and cancer in other tissues. Since so far no association to HPV-related cancer has been reported, these novel upstream regulators may play a central role in HPV-driven cancer development. Remarkably, among the downregulated genes assessed by RNA-Seq (Fig. 5, blue boxes), the five predicted upstream regulators, *STAT1*, *ISG15*, *USP18*, *TGM2*, and *EIF2AK2*, themselves are shown to be affected by multiple other upstream regulators (Fig. 5, blue boxes with yellow frames). This further highlights a strong link between the identified regulatory factors and their activity in response to HPV16 oncogene expression. Since the predictions made by IPA are based on RNA-Seq, selected genes from Fig. 5 were validated by qPCR. The chosen genes again show changes similar to those seen in RNA-Seq (Fig. S2).

10.1128/mSphere.00129-19.2 FIG S2 qPCR validation of selected upstream regulators and target genes. Bar charts are results from qPCR analysis using *TOP1* expression as an internal control. The NOKs pWPI samples were set to a baseline value of 1, to which all gene levels were normalized. Error bars refer to standard deviations for three independent experiments. Statistical analysis was done by unpaired *t* test with Welch’s correction. *, *P* < 0.05; ***, *P* < 0.001; ****, *P* < 0.0001. Download FIG S2, PDF file, 0.6 MB. Copyright © 2019 Yang et al. 2019 Yang et al. This content is distributed under the terms of the Creative Commons Attribution 4.0 International license.

### Genes regulated by identified regulators are involved in multiple biofunctions and cellular pathways.

**FIG 6:**
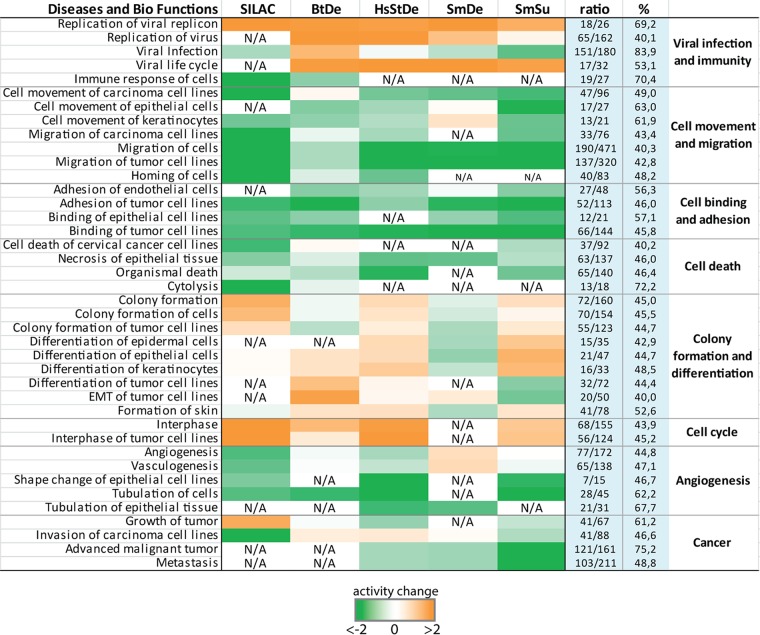
Predicted diseases and biological functions based on genes affected by upstream regulators in response to HPV oncogene expression. Genes that are regulated by predicted upstream regulators in the individual data sets were subjected to comparative pathway analysis using IPA. Predicted effects on the highlighted diseases and biological functions in each group are represented by color. Orange, increased activity; green, decreased activity; N/A, not represented in the respective data set. The ratios (also shown as percentages) depict how many of the DE genes/proteins that were assigned to the respective pathway by IPA were regulated by one or more of the upstream regulators in Fig. 4.

Based on the data sets analyzed by IPA, putatively altered biological functions that can uncover the possible impact of the HPV-induced changes on host cells were revealed. We chose predicted cellular processes that appeared in at least two of the analyzed lists and grouped them according to their functional context (Fig. 6). As expected, in cells expressing HPV oncogenes, processes favoring viral replication and propagation as well as host cell cycle were activated, whereas functions associated with immune responses were inhibited. Note that also cell movement, cell adhesion, and cell death were predicted to be repressed throughout all five lists. The overall tendencies of increased or decreased activity were highly comparable throughout all different data sets. However, some processes, such as colony formation and cell invasion, showed opposing activity changes. This indicates that a slightly different composition of the altered genes can easily tip the balance in opposing directions with respect to such predictive analyses.

We next assessed how many of the DE genes underlying each depicted cellular process from Fig. 6 were reported to be regulated by one or more of the 44 predicted upstream regulators (Fig. 4). The ratios listed for each biological function in Fig. 6 are derived from the number of factors regulated by any of the identified upstream regulators versus the total number of DE genes/proteins assigned to the respective pathway. These ratios, ranging from 40% (migration of cells, epithelial-mesenchymal transition of tumor cell lines) to up to 83% (viral infection), suggest that these regulators play a paramount role in HPV16 *E6E7*-expressing cells.

To further evaluate the significance of the 586 factors (Table S4) that are controlled by the 44 identified upstream regulators, we subjected these genes to pathway enrichment analysis by REACTOME (56). The top 25 predicted pathways are shown in Table 1. Here, seven of the pathways are connected to innate immune signaling, highlighting a major role of HPV oncogene expression to circumvent immunological detection. Apart from mitosis and cell cycle progression, a senescence-associated secretory phenotype was also identified. Moreover, 11 pathways associated with cellular organization, cell contact, and motility, such as extracellular matrix (ECM) composition, collagen synthesis, and integrin and syndecan interactions, were identified. Hence, expression of *E6* and *E7* might affect not only cellular shape and organization but also interactions with surrounding tissues by alterations in the ECM.

**TABLE 1:** Top 25 pathways predicted to be associated with factors affected by upstream regulators^*a*^

Pathway name	No. of found entities/total	Entity ratio	Entity *P* value	FDR	No. of found reactions/total	Reaction ratio
Interferon alpha/beta signaling	66/184	0.013	1.11e–16	3.65e–14	19/20	0.002
Interferon signaling	95/388	0.028	1.11e–16	3.65e–14	45/51	0.004
Interferon gamma signaling	54/250	0.018	1.11e–16	3.65e–14	10/15	0.001
Cytokine signaling in immune system	166/1,051	0.076	1.11e–16	3.65e–14	184/624	0.054
Interleukin-4 and interleukin-13 signaling	46/211	0.015	1.33e–15	3.50e–13	17/46	0.004
Extracellular matrix organization	57/329	0.024	8.55e–15	1.87e–12	221/318	0.027
Immune system	218/2,638	0.19	3.90e–13	7.33e–11	479/1,470	0.126
Nonintegrin membrane-ECM interactions	21/61	0.004	2.67e–11	4.37e–09	13/22	0.002
TP53 regulates transcription of cell death genes	24/83	0.006	3.57e–11	5.21e–09	54/68	0.006
TP53 regulates transcription of death receptors and ligands	12/18	0.001	3.73e–10	4.89e–08	7/7	6.01e–04
Signaling by interleukins	72/640	0.046	1.44e–09	1.72e–07	124/491	0.042
Laminin interactions	14/31	0.002	1.88e–09	2.05e–07	15/15	0.001
Integrin cell surface interactions	22/86	0.006	2.14e–09	2.16e–07	45/54	0.005
Collagen formation	23/104	0.007	1.34e–08	1.26e–06	56/77	0.007
Syndecan interactions	12/29	0.002	6.81e–08	5.93e–06	8/15	0.001
Senescence-associated secretory phenotype (SASP)	20/89	0.006	9.07e–08	7.44e–06	17/22	0.002
Cell cycle	69/681	0.049	1.72e–07	1.32e–05	283/423	0.036
Cell cycle, mitotic	60/569	0.041	3.18e–07	2.24e–05	219/334	0.029
ECM proteoglycans	18/79	0.006	3.24e–07	2.24e–05	17/23	0.002
Assembly of collagen fibrils and other multimeric structures	16/67	0.005	7.93e–07	5.15e–05	25/26	0.002
MET promotes cell motility	13/45	0.003	1.10e–06	6.82e–05	8/12	0.001
Degradation of the extracellular matrix	24/148	0.011	1.67e–06	9.84e–05	55/105	0.009
Endosomal/vacuolar pathway	17/82	0.006	2.39e–06	1.36e–04	3/4	3.44e–04
Collagen biosynthesis and modifying enzymes	16/76	0.005	3.89e–06	2.10e–04	31/51	0.004
Mitotic G_1_ and G_1_/S phases	25/173	0.012	7.35e–06	3.82e–04	57/98	0.008

a Five hundred eighty-six factors regulated by identified upstream regulators were subjected to pathway analysis using the REACTOME database. Found entities are numbers of molecules of the type selected with result types that are common between the submitted data set and the pathway named. The entity ratio is the ratio of entities from this pathway that are molecules of the type selected with “results type” versus all entities in REACTOME of the type selected with “results type.” The entity *P* value is the result of the statistical test for overrepresentation for molecules of the result types selected. The entity FDR is the overrepresentation probability corrected for the false-discovery rate. The number of found reactions is the number of reactions in the pathway that are represented by at least one molecule in the submitted data set for the molecule type selected with “results type.” The reaction ratio is the ratio of reactions from this pathway that contain molecules of the type selected with “results type” versus all REACTOME reactions that contain molecules of the type selected with “results type.”

### Identification of novel factors and gene families potentially involved in carcinogenesis.

Besides identifying the newly identified upstream regulators and the corresponding cellular networks, this study identified several genes and gene families that could not be assigned to any previously reported network or pathway but might be of potential importance in cancer development (Table 2). For example, *CPPED1*, *GPRC5A*, and *TAGLN*, recently reported to have tumor-suppressive function (57–59), were significantly downregulated at both the mRNA and protein level. Conversely, *AURKB*, *KYNU*, and *LCP1* were found to be upregulated by HPV16 E6 and E7. Of note, Aurora kinase B, one of the essential kinases for cell division via regulating mitosis, is also upregulated in multiple cancer types, and its overexpression leads to unequal distributions of genetic information and, subsequently, aneuploidy (60).

**TABLE 2:**
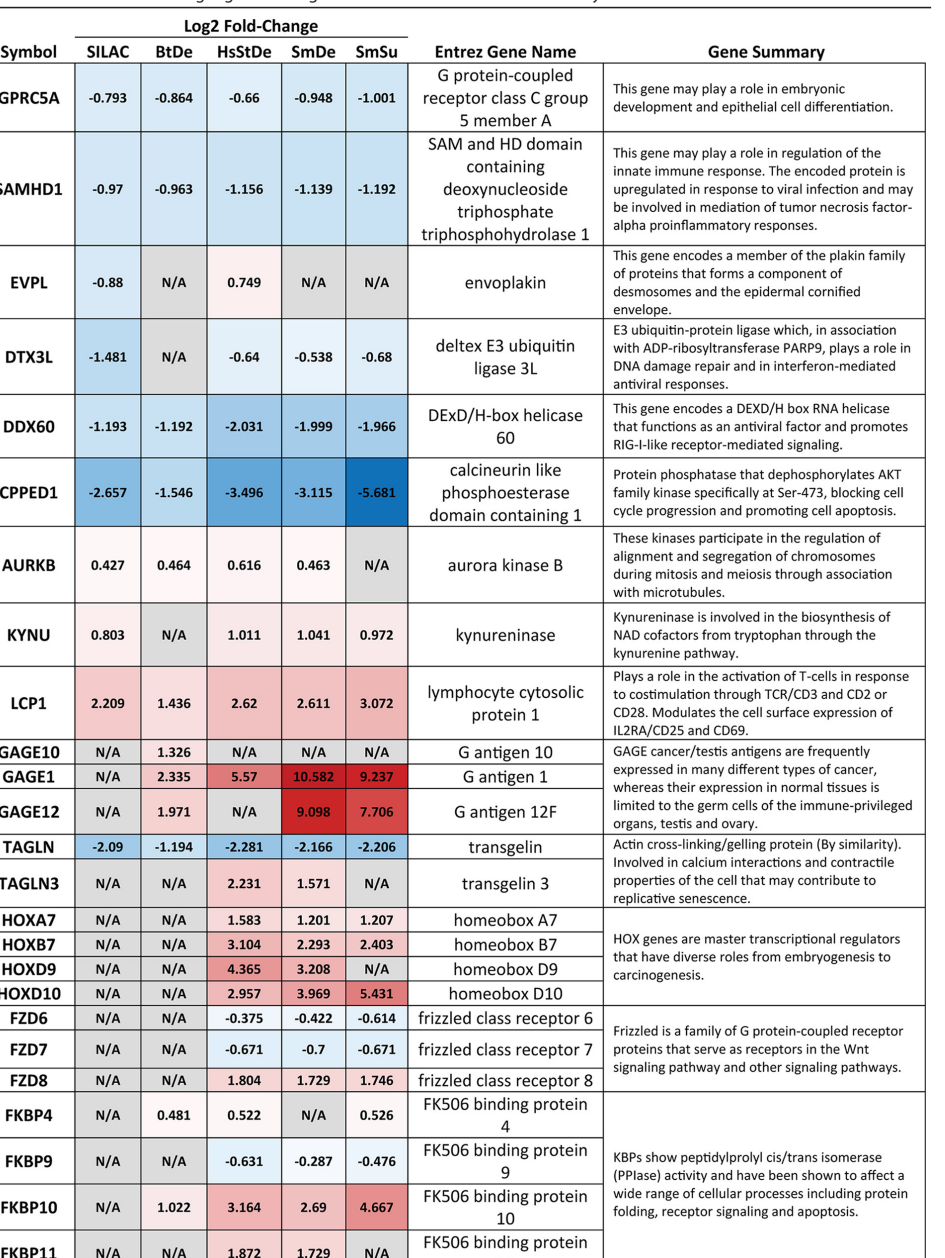
Selected altered single genes and gene families with functional summary^*a*^

a SILAC and RNA-Seq results of selected altered single genes and gene families are shown with log_2_ fold changes. Red, upregulated; blue, downregulated; gray, not available in the respective data set. Gene summaries were conducted from Entrez Gene Summary, CIViC summary, and UniProtKB/Swiss-Prot listed in Genecards.

We also found a deregulation of several gene families, including HOX genes, which are master transcriptional regulators with diverse roles in carcinogenesis (61), frizzled genes, a family of G protein-coupled receptor proteins that serve as receptors in the Wnt signaling pathway (62), and FK506 binding proteins, which have been shown to affect a wide range of cellular processes, including protein folding, receptor signaling, and apoptosis (63). Interestingly, the *GAGE* family, including *GAGE1*, *GAGE10*, and *GAGE12*, are the most strongly upregulated genes found in our study. The *GAGE* gene family codes for a group of cancer-testis antigens (CT antigens) that is expressed only in testis under normal conditions but widely expressed in multiple types of cancer tissue (64).

### TCGA analysis of newly identified upstream regulators and single factors: clinical relevance.

The focus of our study was ultimately the identification of novel factors whose alterations can be seen both at early stages of transformation (represented by our model) and in actual patient-derived tumors, indicating a protumorigenic association. For this purpose, we chose several of the newly identified upstream regulators, their downstream targets, and some markedly changed single factors to examine their expression levels in RNA-Seq data from The Cancer Genome Atlas (TCGA). Besides cervical cancer (CESC) data sets, data sets for head and neck squamous cell carcinoma (HNSC), esophageal cancer (ESCA), different urogenital cancers (uterine, ovarian, and rectal carcinoma), and lung squamous cell carcinoma (LUSC) were included in order to evaluate our findings with respect to pan-cancer or HPV-specific aberrations.

**FIG 7:**
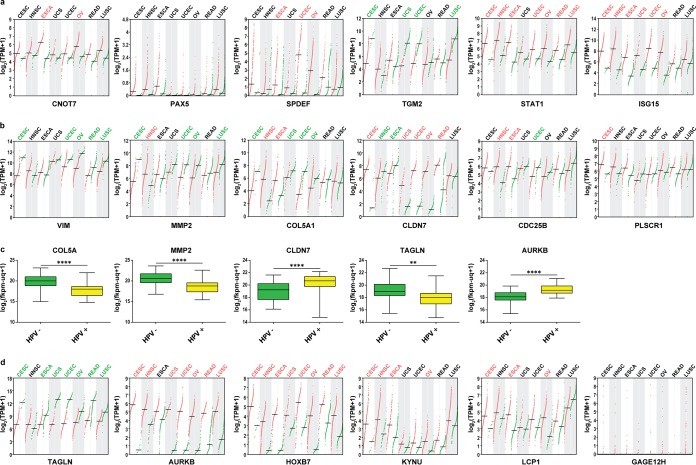
Expression profiling of selected genes in TCGA RNA-Seq data sets. Profiling plots show the expression of distinct genes in multiple cancer types. Differential analysis was done by GEPIA using TCGA tumor samples with paired adjacent TCGA normal samples and GTEx normal samples. The log_2_(TPM plus 1) values of tumor samples are plotted in red, and the values from normal tissue samples are plotted in green. Cancer type abbreviations shown in red and green suggest significant (*q *<* *0.01) up- or downregulation of the mentioned genes in tumor samples. Four-way ANOVA, using sex, age, ethnicity, and disease state (tumor or normal tissue) as variables, was used for calculating differential expression. TPM, transcripts per million; CESC, cervical squamous cell carcinoma and endocervical adenocarcinoma; HNSC, head and neck squamous cell carcinoma; ESCA, esophageal carcinoma; UCS, uterine carcinosarcoma; UCEC, uterine corpus endometrial carcinoma; OV, ovarian serous cystadenocarcinoma; READ, rectum adenocarcinoma; LUSC, lung squamous cell carcinoma. (a) Profiling plots of upstream regulators identified in this study. (b) Profiling plots of selected target genes of predicted upstream regulators. (c) Box plots show genes that are differentially expressed in HPV-positive HNSCs from those in HPV-negative HNSCs. Analysis was done with the UCSC Xena browser using GDC TCGA head and neck cancer data sets. HPV status was determined by p16 testing. The log_2_(fkpm-uq plus 1) values, where fkpm-uq is the number of fragments per kilobase of transcript per million mapped reads upper quartile, of all samples were used for differential analysis. HPV-positive HNSCs, *n* = 31; HPV-negative HNSCs, *n* = 74. Statistics was done by unpaired *t* tests with Welch’s correction. **, *P* < 0.001; ****, *P* < 0.00001. (d) Profiling plots of isolated genes whose expression showed significant changes in our RNA-Seq analysis.

Figure 7a shows the RNA-Seq profiling of six identified upstream regulators from Fig. 5 in the above-mentioned cancer entities. The mean levels of *CNOT7*, *PAX5*, and *SPDEF* are slightly elevated in CESC samples compared with those in healthy tissue, though not significantly, while the level of *TGM2* is significantly downregulated in cervical cancer. One possible explanation for this is that the prediction of the activation status of the upstream regulators may not necessarily be associated with the mRNA level. In the case of *TGM2*, it not only was predicted to be downregulated as an upstream regulator but also showed decreased mRNA levels, which is consistent with data from cancer patients. Remarkably, *STAT1*, which has been intensively reported by numerous studies to be downregulated by hrHPV oncogenes, is strongly upregulated not only in CESC but also in the majority of cancer types listed here. Similar results were observed for *ISG15*.

Further, we selected several genes that were predicted to be targets of the identified upstream regulators (Fig. 7b). *VIM*, *MMP2*, and *COL5A* are significantly downregulated in CESC, while *CLDN7* is upregulated, which is consistent with our study. The levels of *CDC25B* and *CCND1*, however, are barely changed in CESC compared to levels in normal tissue. Interestingly, we noticed that the expression of *MMP2*, *COL5A*, and *CLDN7* showed completely different tendencies in CESC and HNSC. While more than 90% of cervical squamous cell carcinomas are found to be HPV positive, the prevalence of HPV substantially varies for HNSC (65). We therefore analyzed the levels of *MMP2*, *COL5A1*, and *CLDN7* in HPV-positive and HPV-negative HNSC samples to determine whether HPV status plays a role in the expression of these genes (Fig. 7c). For both *MMP2* and *COL5A*, the mRNA levels in HPV-positive tumor samples were significantly reduced, whereas the levels of *CLDN7* were increased. This finding indicates that depending on the HPV status of HNSC tumors, expression of these genes is either comparable or contrary to their expression in cervical cancers, suggesting that at least for *MMP2*, *COL5A1*, and *CLDN7*, the transcriptional changes might be dependent on HPV *in vivo*.

Finally, *TAGLN* (Fig. 6), which was identified as an important downregulated factor with a potential tumor suppressor function, is significantly downregulated in almost all cancer types listed here (Fig. 7d). *AURKB*, *HOXB7*, *KYNU*, and *LCP1* are strongly upregulated in CESC, with the first two also upregulated in multiple other cancer types. The expression of *TAGLN* and *AURKB* was also analyzed in HPV-positive and HPV-negative HNSC samples (Fig. 7c). In HPV-positive HNSC, the expression of *TAGLN* is significantly decreased, while *AURKB* is strongly upregulated, suggesting that HPV might be responsible for gene alterations that are important in cancer development in general. *GAGE12H* was found to be strongly upregulated in our system; however, TCGA data show that throughout the tumor samples, there are only a few which display a strong upregulation of *GAGE12H* across all cancer types, indicating random or patient-dependent alterations.

## DISCUSSION

Although the impact of HPV infection on host cells has been studied intensively, the mechanisms underlying virus-induced transformation are still not completely understood. The vast number of host factors that are deregulated in response to viral gene expression makes reliable model systems indispensable for a bottom-up analysis to further investigate *in vitro* findings with clinical relevance at a mechanistic level. Depending on the differentiation status of the host cell and the viral life cycle, tremendous differences in transcriptome and proteome profiles can be expected and have to be taken into account when choosing a model system. Several omics studies were performed with either freshly infected keratinocytes in a raft system (26) or long-term adapted cervical carcinoma lines obtained from patients (22, 66). In general, viral integration, which precedes malignant transformation, always abolishes the productive virus life cycle. Subsequently, the predominant expression of the oncogenes *E6* and *E7* becomes the main driving force behind host cell transformation. Therefore, we used freshly established HPV16 *E6*- and *E7*-expressing immortalized human oral keratinocytes and combined quantitative proteome and transcriptome analyses (Fig. 1 and 2) to provide a more complete view on the impact of HPV oncogene expression on host cells preceding and putatively contributing to tumorigenesis.

We followed the workflows outlined in Fig. 2, and 290 proteins were found to be significantly altered in HPV-positive cells; of these, 155 were also differentially expressed in RNA-Seq analyses (Fig. 3). This suggests a substantial role of transcriptional regulation in the cellular changes caused by HPV16 E6E7. Alterations of protein levels that are not reflected in RNA-Seq analyses might be the result of either low mRNA steady-state levels, with fluctuations diminishing significance, or posttranslational modifications.

One major challenge in such global approaches is to find common central upstream regulatory networks that ultimately control clusters of downstream genes. Pathway analysis platforms like IPA provide a solution to this aim by putting data into the context of previous and current research. Based on DE genes identified here, IPA predicted marked activity changes in 44 putative upstream regulators (Fig. 4) that were associated with 14 to 32% of all deregulated factors (see Table S3 in the supplemental material). For instance, while MITF, SPDEF, CNOT7, BTK, PAF1, and PAX5 seem to be novel key regulators in the context of HPV-induced carcinogenesis, they are apparently already known to play essential roles in other cancer entities (Fig. 4). This is in agreement with the concept of multistep carcinogenesis stating that tumor viruses mostly target regulatory proteins that are also functionally affected in tumors without any viral etiology (12). Tumorviruses as evolutionary sophisticated entities—independently of their origin and tissue tropism—always attack central hubs of a cellular network, a concept referred to as “local-impact hypothesis” (49).

Many of the identified upstream regulators are directly or indirectly controlled by each other, revealing a network with a hierarchical structure (Fig. 5). Within this network, the transcription factors MITF, SPDEF, and CNOT7 stand out because they are predicted to directly regulate other cellular key factors, like STAT1 and HIF1α (55). Specifically, CNOT7 binds and inhibits the antiproliferative protein BTG1, downregulates innate immune responses (91), and serves as an upstream regulator of STAT1 (54). SPDEF is usually expressed in prostate epithelium, and higher expression levels are associated with prostate cancer and cancers of the brain, lung, breast, and ovaries (67). MITF regulates melanocyte differentiation and is known to activate genes with essential roles in cell differentiation, proliferation, and survival, which is also the case in our analysis (Fig. 5a) (68). The last two findings are interesting because these genes are usually expressed in cell types other than keratinocytes and may therefore represent promising candidates for further studies in the context of HPV-induced carcinogenesis. Of important note is that several of the connections identified by IPA are based on single reports in only one cell line. It is therefore indispensable to view these predicted associations and regulations with caution when considering them for future studies.

Concerning the biological status of cells expressing HPV16 oncogenes, pathway analysis (Fig. 6 and Table 1) showed that, apart from known physiological changes, like a decrease in innate immune responses and increased cell cycle progression and differentiation, pathways associated with cell-cell contact and movement were especially enriched, a finding that is consistent with a recent study using raft cultures with cervical keratinocytes (50). Cell adhesion and cell migration were predicted to be decreased to similar extents, which is possibly rooted in the fact that genes involved in these pathways show a strong overlap. Furthermore, oncogene expression is predicted to affect cell shape and possibly favor epithelial mesenchymal transition (EMT) at later stages of virus-induced transformation. Of special interest is the additional prediction of a senescence-associated secretory phenotype (SASP). The SASP has been shown to play a paradoxical role in cancer formation. Recent studies showed that it contributes to tumor-promoting inflammation, an immunosuppressive microenvironment, and EMT, thus acting as a driving force of malignancy in premalignant cells (69–72).

We identified genes and gene families that might inhibit or drive virus-induced carcinogenesis that are distinct from highly interactive networks of factors (Table 2; Fig. S1b). CPPED1, also known as CSTP1, has been reported to be involved in blocking the cell cycle, promoting apoptosis, and suppressing tumor growth as a potential tumor suppressor (57). Its downregulation, furthermore, leads to improved glucose metabolism (73). Here, CPPED1 was dramatically downregulated (up to a log_2_ fold change of less than −5) at both the mRNA and protein level. Similarly, GPRC5A and TAGLN, two reported potential tumor suppressors in lung cancer (58, 59), were also observed to be downregulated after expression of HPV16 oncogenes. While it is known that HPV16 can modulate host tryptophan metabolism, a key metabolic event contributing to immune escape by upregulating the immunoregulatory molecule indoleamine 2,3-dioxygenase (IDO 1) (74), much less is known about the role of kynureninase (KYNU), another key enzyme in the kynurenine pathway. LCP1, a member of the actin-binding protein family of plastins is important for the activation of human peripheral blood T lymphocytes (75) and was recently reported to be upregulated and to serve a critical role in oral squamous cell carcinomas (76). The upregulation of factors like LCP1 (usually expressed in lymphocytes) and members of the GAGE cancer/testis antigen family are intriguing. Aberrant occurrences of such factors in keratinocytes can putatively serve as diagnostic markers or therapeutic targets when they are expressed in tumor tissues, which has recently been tried in GAGE-expressing cancers (64).

To assess the clinical relevance of our observed findings, we chose several validated factors from Fig. 5 and Table 2 and compared them to transcriptome data from TCGA (Fig. 7). We tried to provide a comparison of our results with patient data in order to identify factors that have not previously been described in the context of HPV oncogene expression but were also frequently altered in tumors of different origins, defining them as likely candidates promoting tumorigenesis. Here, the same tendency in expression changes of *TGM2*, *MMP2*, *CLDN7*, *HOXB7*, and *LCP1* can be found in different cancer types, including cervical and head and neck squamous cell carcinoma. However, not all selected DE genes were significantly altered in patients. Immunity-associated genes, like *STAT1*, whose downregulation by HPV has been reported (77, 78), were found to be upregulated in almost all analyzed cancer tissues. The virus-induced inhibition of the immune system is a known prerequisite for viral persistence, and immunological surveillance also strongly depends on the anatomic site (30). After the productive life cycle is aborted upon viral integration, cells that undergo malignant transformation can subsequently display a chronic inflammatory phenotype, which can be seen as a general hallmark of cancer (17). Furthermore, network interactions can also be affected by the amount of E6/E7 oncoproteins within the respective host cell and may explain why transcriptomes of infected cells with high copy numbers differ from host cells, harboring only a few copies of integrated HPV (25, 26). Another possible explanation for genes with inconsistent changes within TCGA data might be due to variations among individuals. This is prominently visible for *GAGE12H*, which was upregulated by several hundredfold in our study but is not significantly altered in data from TCGA (Fig. 7d). Its expression is strongly increased in some tumors, whereas in others, the mRNA levels are nearly undetectable. Factors like these might therefore be interesting candidates in personalized medicine.

Notably, several genes whose expression changes in our *in vitro* model also appear to be linked to HPV positivity in patient samples (Fig. 7c). Upon comparison of CESC and HNSC, an opposing change in the expression levels of certain genes was apparent. In contrast to HPV-negative HNSC samples, HPV-positive tumors showed the same tendency as cervical cancers with respect to the expression of *COL5A*, *MMP2*, *CLDN7*, *TAGLN*, and *AURKB*, supporting their possible contribution to virus-induced carcinogenesis. This is consistent with a previous study where the transcriptomes of more than 4,400 tumors and 19 different types of cancer were analyzed (79). For instance, *TAGLN* is a tumor suppressor that is downregulated in multiple cancers, and its loss seems to be an early event in cell transformation, coinciding with alterations in cellular plasticity (80). The latter fits the numerous changes that we observed in factors involved in cell shape and organization, as well as cell-cell contact, including *COL5A*, *MMP2*, and *CLDN7*. Upregulation of *CLDN7* has been found in various types of cancer, suggesting that its deregulation by HPV might be part of an early event during cell transformation (81, 82).

In conclusion, using a highly integrative approach allowed the identification of novel HPV-induced cellular changes that are also reflected in cancer patients, providing a basis for future studies in both basic and translational research.

## MATERIALS AND METHODS

### Cell culture.

NOKs pWPI and NOKs HPV16 E6E7 were kept in keratinocyte serum-free medium (SFM) (Life Technologies) containing recombinant human epidermal growth factor (rhEGF) and bovine pituitary gland extract (Life Technologies) for daily cultivating and passaging. Cells were switched into flavin adenine dinucleotide (FAD) medium containing 75% Ham’s F-12 medium, 25% Dulbecco’s modified Eagle’s medium (DMEM), 5% fetal bovine serum (FBS), insulin (5 μg/ml), epidermal growth factor (10 ng/ml), cholera toxin (8.4 ng/ml), adenine (24 μg/ml), and hydrocortisone (0.4 μg/ml) for one passage before being used for experiments. HEK293T cells were grown in high-glucose DMEM (Sigma-Aldrich) supplemented with 10% FBS (Gibco). The HPV16-positive cervical carcinoma cell line CaSki (ATCC CRL-1550 was maintained according to instructions from the American Type Culture Collection [ATCC]).

### Cloning of expression plasmids.

pWPI-Puro was generated by replacing the green fluorescent protein (GFP) in pWPI (Addgene catalog number 12254) with a puromycin resistance sequence. The P2A sequence of porcine teschovirus-1 was synthesized and cloned into pWPI-Puro after two rounds of overhang PCR to create a GSG linker and necessary restriction sites for further cloning. The sequences of all cloning primers are listed in Table S1 in the supplemental material. HPV16 *E6* was subcloned from plasmid MSCV-IP N-HA 16E6 (Addgene catalog number 42603; gift from Peter Howley) into pCMV-3Tag-1A (Agilent) with Phusion polymerase (Thermo Fisher Scientific) using the primers 16E6 pCMV-F and 16E6 pCMV-R to generate 3×FLAG-tagged HPV16 *E6*. Tagged HPV16 *E6* was further cloned into pWPI-Puro-P2A using the primers 3Flag-P2A-pWPI-F and 16E6-3Flag-P2A-pWPI-R. HPV16 *E7* was amplified from MSCV-P C-FlagHA 16E7-Kozak (Addgene catalog number 35018; gift from Peter Howley) and tagged by Twin-Strep-tag using five rounds of overhang PCR with the primers 16E7-Strep-P2A-pWPI-F and 16E7-Strep-P2A-pWPI-R1, Strep-P2A-pWPI-R2, Strep-P2A-pWPI-R3, Strep-P2A-pWPI-R4, or Strep-P2A-pWPI-R5. Twin-Strep-tag-tagged *E7* was further cloned into pWPI-Puro-P2A. Plasmids were amplified in One Shot Stbl3 chemically competent Escherichia coli (Thermo Fisher Scientific).

10.1128/mSphere.00129-19.3 TABLE S1 Oligonucleotides employed in this study. Download Table S1, DOCX file, 0.02 MB. Copyright © 2019 Yang et al. 2019 Yang et al. This content is distributed under the terms of the Creative Commons Attribution 4.0 International license.

### Lentivirus production and transduction of NOK cells.

HEK293T cells were cotransfected by lentiviral expression plasmids, packaging plasmid psPAX2 (Addgene catalog number 12260), and enveloping plasmid pMD2.G (Addgene catalog number 12259; gift from Didier Trono) using 25-kDa linear polyethylenimine (Polysciences). Forty-eight hours after transfection, the supernatant was collected, filtered, and applied to NOKs in the presence of 10 µg/ml Polybrene (Santa Cruz). Virus-containing medium was removed after 24 h, and cells were kept in medium supplemented with 1 µg/ml puromycin (Sigma-Aldrich) for selection until all untransduced control cells were dead. Only cells within 10 passages after selection were used for experiments.

### Protein extraction and Western blotting.

Monolayer cells were washed with 1× phosphate-buffered saline (PBS), harvested in 1.25× Laemmli buffer (78 mM Tris, pH 6.8; 2.5% SDS; 6.25% glycerol; 0.125% bromophenol blue; 2.5% β-mercaptoethanol), and immediately incubated at 99°C for 5 min and then placed on ice. Samples were treated with 100 U/ml Benzonase (Millipore) for 5 min at room temperature, and protein concentration was determined with a NanoDrop spectrophotometer (Thermo Fisher Scientific). Either 30 or 50 µg protein (as indicated in the figure legends) per lane was applied for Western blotting with the following antibodies: anti-FLAG M2 (Sigma-Aldrich catalog number F3165), anti-Strep-tag (IBA catalog number 2-1509-001), anti-p53 DO-1 (Santa Cruz Biotechnology catalog number sc-126), anti-pRb 4H1 (Cell Signaling Technology catalog number 9309), anti-HPV16 E7, clone NM2 (gift from Martin Müller, DKFZ), anti-HPV16 E6 (Euromedex catalog number E6-6F4), anti-kynureninase E-5 (Santa Cruz Biotechnology catalog number sc-390360), anti-CPPED1 H-11 (Santa Cruz Biotechnology catalog number sc-514222), anti-l-Plastin B-9 (Santa Cruz Biotechnology catalog number sc-133218), anti-transgelin 6G6 (Santa Cruz Biotechnology catalog number sc-53932), and goat-anti mouse IgG (H+L) horseradish peroxidase (HRP; Jackson ImmunoResearch).

### RNA extraction and reverse transcription.

RNA was extracted using the RNeasy minikit (Qiagen) according to the manufacturer's instructions. RNase-free DNase Set (Qiagen) was used to remove residual DNA for downstream applications. RNA concentrations were determined by NanoDrop spectrophotometry, and 1 µg of RNA was reverse transcribed with a RevertAid real-time reverse transcription kit (Thermo Fisher Scientific) and oligo(dT)_22_ primers (Thermo Fisher Scientific) according to the manufacturer's instructions.

### PCR.

Semiquantitative RT-PCRs were performed using DreamTaq Green DNA polymerase (Thermo Fisher Scientific) according to the manufacturer's instructions. Quantitative real-time PCR (qPCR) was performed on a CFX96 Touch real-time PCR detection system (Bio-Rad) using iTaq Universal SYBR Green supermix (Bio-Rad) according to the manufacturer's instructions. Samples used for qPCR were generated from cells seeded on three different days. The sequences of all primers used in this study are listed in Table S1.

### RNA sequencing.

RNA samples used for sequencing were harvested from cells seeded on three different days. A TruSeq Stranded mRNA library prep kit (Illumina) was used for mRNA enrichment and generation of 50-bp cDNA libraries for single-read sequencing with Illumina HiSeq 2000 v4 (DKFZ Genomics and Proteomics Core Facility) according to the manufacturer’s recommendations. A depth of 30 to 40 million reads was reached for each sequenced sample.

### RNA-Seq data analysis.

For all samples, low-quality bases were removed using Fastq_quality_filter from the FASTX Toolkit (v0.0.13, http://hannonlab.cshl.edu/fastx_toolkit/index.html), with 90% of the reads requiring a Phred quality score of >20. Homertools 4.7 (83) was used for poly(A) tail trimming. Reads with a length of <17 bp were removed. PicardTools (v1.78, https://broadinstitute.github.io/picard/) was used to compute the quality metrics with CollectRNASeqMetrics. For BtDe (bowtie2 plus DESeq2), mapping was carried out with bowtie2 (v2.2.4) (84) against union human genes: every gene is represented by a union of all its transcripts (exons). The count values (transcripts per million [TPM] and raw counts) were calculated by running CoverageBed from Bedtools (v2.17.0) (92) of the mapped reads together with a specific annotation file for protein-coding genes (based on Ensembl 85) in gtf format and by parsing the output with custom Perl scripts. For DESeq2 (85), DESeqDataSetFromMatrix was applied, followed by estimateSizeFactors, estimateDispersions, and nbinomWald testing. For HsStDe (HISAT2 plus StringTie plus DESeq2), raw reads were aligned against the human reference genome (assembly GRCh37) using HISAT2 (v2-2.1.0, https://ccb.jhu.edu/software/hisat/index.shtml) (93, 94) with default parameters. Unmapped reads were subsequently mapped to the HPV16 genome (GenBank accession number NC_001526.2). The mapping rate is shown in Table S2 in the supplemental material. StringTie (v1.3.3b, https://ccb.jhu.edu/software/stringtie/index.shtml) (94, 95) and prepDE.py, a special Python script provided with the StringTie tool (https://ccb.jhu.edu/software/stringtie/dl/prepDE.py), were used to assemble the transcripts and extract read count information for DESeq2. The read count matrix was processed by DESeq2 (v1.16.1, https://bioconductor.org/packages/release/bioc/html/DESeq2.html) for differential expression analysis. For SmSu (Salmon plus sleuth), sequences were pseudoaligned to the human transcriptome (Ensembl 85 version) with Salmon (v0.8.2) (96) in quasi-mapping mode using bootstraps to compute abundance estimates. Salmon output was converted by wasabi (https://github.com/COMBINE-lab/wasabi) into the format which is compatible with sleuth (86) for differential-expression analysis. Differential expression was assessed using sleuth (v0.29.0) with the Wald test to determine differentially expressed (DE) transcripts. For SmDe (Salmon plus DESeq2), Salmon output was imported to DESeq2 by tximport function (https://bioconductor.org/packages/release/bioc/html/tximport.html) to quantify gene expression levels. In all the workflows mentioned above, genes with *q* values of <0.05 were considered DE genes with statistical significance.

10.1128/mSphere.00129-19.4 TABLE S2 Overview of obtained reads from RNA-Seq analysis mapped to human genome GRCh37 (hg19) and the HPV16 genome. Download Table S2, DOCX file, 0.02 MB. Copyright © 2019 Yang et al. 2019 Yang et al. This content is distributed under the terms of the Creative Commons Attribution 4.0 International license.

10.1128/mSphere.00129-19.5 TABLE S3 List of factors whose levels changed significantly at both the mRNA and protein level upon HPV oncogene expression. Numbers represent log_2_ fold changes for each factor obtained by the different workflows. N/A, changes in this gene had a *q* value of >0.05 or were not detected in the respective data set. Download Table S3, DOCX file, 0.07 MB. Copyright © 2019 Yang et al. 2019 Yang et al. This content is distributed under the terms of the Creative Commons Attribution 4.0 International license.

### Cell labeling, lysis, and protein digestion for SILAC.

The proteomic data were achieved in a larger and integrated experiment involving comparison of NOKs pWPI, NOKs HPV16 E6E7, and NOKs HPV16 E6E7 infected with Chlamydia trachomatis. For the purpose of this study, only the data comparing NOKs HPV16 E6E7 to NOKs pWPI were further analyzed. SILAC FAD media were prepared from DMEM for SILAC (Thermo Fisher Scientific) and Ham’s F-12 medium for SILAC (Thermo Fisher Scientific) supplemented with either l-arginine-HCl plus l-lysine–2HCl (light), l-arginine–HCl (^13^C_6_) plus l-lysine–2HCl (4,4,5,5-D_4_) (medium), or l-arginine–HCl (^13^C_6_, ^15^N_4_) plus l-lysine–2HCl (^13^C_6_, ^15^N_2_) (heavy). Cells were grown in SILAC FAD media for 8 days to reach a labeling efficacy of >99.9%, while the proline conversion rate was <4%. Cell pellets were suspended in 1% (wt/vol) sodium deoxycholate in 50 mM ammonium bicarbonate, boiled at 99°C for 10 min, and cooled to 4°C. The lysates were then treated with benzonase (Sigma-Aldrich) in a final concentration of 150 U/ml for 60 min on ice. Insoluble material was removed by centrifugation at 14,000 × *g* for 15 min at 4°C. Protein concentration was determined by a modified bicinchoninic acid (BCA) assay (87). Corresponding light, medium, and heavy lysates were mixed 1:1:1 (protein ratio), and mixed samples were diluted with 50 mM ammonium bicarbonate to adjust the protein concentration to 0.5 µg/µl. Lysates were then reduced with 10 mM dithiothreitol at 37°C for 60 min and alkylated with 20 mM iodoacetamide at room temperature for 30 min in the dark, and the unreacted iodoacetamide was quenched with further 10 mM dithiothreitol at room temperature for 15 min. Proteins were digested with sequencing-grade trypsin (Promega) overnight at 37°C. Sodium deoxycholate was removed by the modified phase transfer protocol (88).

### Peptide separation and MS analysis.

Peptides were separated by two-dimensional liquid chromatography (LC). In the first dimension, a manual chromatographic device (97, 98) was used in a modified setting for basic-pH reversed-phase LC fractionation of the complex peptide samples. Briefly, the microcolumn (0.25 by 30 mm) was packed with 2.6-µm Kinetex EVO C_18_ core shell particles (Phenomenex) in a fluorinated ethylene propylene tubing (VICI Jour). Thirty micrograms of peptide mixture dissolved in 6 µl of 2% acetonitrile (ACN)-0.1% trifluoroacetic acid (TFA) was loaded on the microcolumn using a gastight microsyringe. The peptides were eluted over the elution volume of 24 µl by the gradient formed in a microsyringe from mobile phases containing 2%, 8%, 16%, 24%, 32%, and 40% ACN in 20 mM ammonium formate (pH 10). Separated peptides were collected in 13 elution fractions, acidified with 1% TFA, and dried in a vacuum. The first two fractions and the last fraction were combined. The second dimension of the separation was performed on an Ultimate 3000 RSLCnano system (Dionex, USA) coupled on-line through a Nanospray Flex ion source by Q-Exactive mass spectrometry (MS) (Thermo Scientific). Fractions were dissolved in 2% ACN-0.05% TFA and loaded on capillary trap column (C_18_ PepMap100, 3 µm, 100 Å, 0.075 by 20 mm; Dionex) by 5 µl/min of 2% ACN-0.05% TFA for 5 min. Then they were separated on a capillary column (C_18_ PepMap rapid-separation LC, 2 µm, 100 Å, 0.075 by 150 mm; Dionex) by step linear gradient of mobile phase B (80% ACN-0.1% formic acid) over mobile phase A (0.1% formic acid) from 4% to 34% of phase B in 36 min and from 34% to 55% of phase B in 11 min at a flow rate of 300 nl/min. The column was kept at 40°C, and the eluent was monitored at 215 nm. The spraying voltage was 1.75 kV, and the heated capillary temperature was 275°C. The mass spectrometer operated in the positive-ion mode by performing survey MS (at 350 to 1,650 *m/z*) and data-dependent tandem MS (MS/MS) scans on the 10 most intense precursors, with a dynamic exclusion window of 30 s. MS scans were acquired with a resolution of 70,000 from 10^6^ accumulated charges; the maximum fill time was 100 ms. The intensity threshold for triggering MS/MS was set at 5 × 10^4^ for ions with a *z* of ≥2 and an isolation window of 1.6 Da. Normalized collision energy for high-cell-density (HCD) fragmentation was 27 units. MS/MS spectra were acquired with a resolution of 17,500 from 10^5^ accumulated charges; the maximum fill time was 100 ms.

### Protein identification and quantification.

Raw data sets were processed by MaxQuant (v1.6.0.1) coupled with the Andromeda search engine (89). Data were searched against a database constructed by merging the reference proteome sets of Homo sapiens (downloaded from UniProt, accession number UP000005640, November 2017), human papillomavirus types 16 and 18 (accession numbers UP000106302 and UP000009109, respectively, November 2017), and Chlamydia trachomatis (accession number UP000050023, March 2017). The MaxQuant-implemented database was used for the identification of common contaminants. The identification and quantification parameters of MaxQuant were set as follows: the mass tolerance for the first search was 20 ppm, that for the second search from recalibrated spectra was 4.5 ppm (with individual mass error filtering enabled), the maximal charge per peptide (*z*) was 7, the minimal peptide length was 7 amino acids, the maximal mass of the peptide was 4,600 Da, the fixed modification was carbamidomethylation of cysteine, variable modifications were oxidation of methionine and acetylation of the protein N terminus, the maximum number of variable modifications was set to 5 per peptide, and digestion with trypsin/P was with a maximum of 2 missed cleavages. Mass tolerance for fragments in MS/MS was 20 ppm, with the 12 most intensive peaks per 100 Da taken for the search. The minimal Andromeda score for modified peptides was 40, and the minimal delta score for modified peptides was 6. The false-discovery rate (FDR) estimation of peptide identification was based on a target decoy approach using a reverted-search database. An FDR filter of 0.01 was applied to the peptide spectrum match level. For peptide quantitation, Arg plus 6[^13^C_6_] and Lys plus 4[D_4_] were set for the medium channel and Arg plus 10[^13^C_6_^15^N_4_] and Lys plus 8[^13^C_6_^15^N_2_] were set for the heavy channel. The requantify function was enabled. Normalized ratios were used for the calculation of labeling site occupancies. For protein quantification, only protein groups with at least 2 unique peptides with a SILAC ratio were considered. The light, medium, and heavy SILAC labels were swapped to make different combinations in the three replicates. For the purpose of MaxQuant, the fractions from basic-pH LC separation were set as one experiment.

Proteomics data analysis was performed with Perseus (v1.5.3.0) after the protein group files generated by MaxQuant (90) were uploaded. Available annotations were uploaded from the Homo sapiens database (release date, 20 June 2015). Proteins identified only by site and reverse database hits, as well as potential contaminants, were removed. The replicate samples were grouped, and 1/*x* transformation was applied when necessary. The normalized ratios were subsequently log_2_ transformed. Proteins with fewer than two values in at least one group were removed, and missing values were imputed from a normal distribution around the detection limit. A one-sample test was performed. A *P* value smaller than 0.05 was used as a threshold to identify proteins whose levels were significantly altered.

### Network and functional analysis.

The regulatory networks and functional analyses were generated through the use of IPA (Qiagen Inc.).

### Analysis of selected data sets in GEO.

Data from Gene Expression Omnibus (GEO) (accession numbers GSE92496, GSE38467, and GSE109039) were used to generate Table S4 and Table S6 for comparison of studies. For Table S4, the average log_2_ fold change of genes from accession number GSE92496 (duplicates) were calculated and compared to the significantly deregulated gene lists from the present study, accession number GSE38467, and accession number GSE109039. For the analysis of upstream regulators, the activation scores for GSE92496 were taken directly from its publication (48), while it was calculated by the upstream regulator prediction function in IPA for GSE38467 and GSE109039.

10.1128/mSphere.00129-19.6 TABLE S4 The 155 factors whose levels changed significantly at both the mRNA and protein levels upon HPV oncogene expression in this study were compared to those in three previously published studies. HFK, human foreskin keratinocytes; HCK, human cervical keratinocytes; HLF, human lung fibroblasts. Download Table S4, DOCX file, 0.04 MB. Copyright © 2019 Yang et al. 2019 Yang et al. This content is distributed under the terms of the Creative Commons Attribution 4.0 International license.

10.1128/mSphere.00129-19.7 TABLE S5 Numbers of factors affected by 44 putative upstream regulators in the individually obtained data sets. Download Table S5, DOCX file, 0.01 MB. Copyright © 2019 Yang et al. 2019 Yang et al. This content is distributed under the terms of the Creative Commons Attribution 4.0 International license.

10.1128/mSphere.00129-19.8 TABLE S6 Selected predicted upstream regulators that are also supported by similar studies. HFK, human foreskin keratinocates; HCK, human cervical keratonocytes; HLF, human lung fibroblasts. Download Table S6, DOCX file, 0.02 MB. Copyright © 2019 Yang et al. 2019 Yang et al. This content is distributed under the terms of the Creative Commons Attribution 4.0 International license.

### TCGA data analysis.

Differential analysis for selected genes in eight different cancer types was performed by gene expression profiling interactive analysis (GEPIA; http://gepia.cancer-pku.cn/) (99) using log_2_(TPM plus 1) values of TCGA tumor samples with paired adjacent TCGA normal samples and GTEx normal samples. Four-way analysis of variance (ANOVA), using sex, age, ethnicity, and disease state (tumor or normal) as variables, was used for calculating differential expression. Differential-expression analysis for HPV-positive HNSC samples compared to HPV-negative HNSC samples was performed with the UCSC Xena browser (https://xenabrowser.net/) with the GDC TCGA Head and Neck Cancer data set (100). HPV status was determined by p16 testing. The log_2_(fkpm-uq plus 1) values of all samples were used for differential analysis. Statistics was performed by unpaired *t* tests with Welch’s correction.

### Statistics.

Statistics for qPCR results was performed using GraphPad Prism 6 (v6.02) with the unpaired *t* test with Welch’s correction. Perseus software (v1.5.3.0) was used for statistical analysis of proteomic data as indicated above. RNA-Seq statistical analysis was carried out using either DESeq2 or Sleuth as mentioned earlier. For TCGA data, statistics was performed using either GEPIA or the UCSC Xena browser by unpaired *t* tests with Welch’s correction.

### Data availability.

The RNA-Seq data provided in this study have been deposited in NCBI’s Gene Expression Omnibus and are accessible through GEO series accession number GSE124357. The mass spectrometry proteomics data can be accessed at the ProteomeXchange Consortium via the PRIDE (101) partner repository at https://www.ebi.ac.uk/pride/archive/. The data set identifier is PXD012186.
