# Supplementary figures and images for: Combined Transcriptome and Proteome Analysis of Immortalized Human Keratinocytes Expressing Human Papillomavirus 16 (HPV16) Oncogenes Reveals Novel Key Factors and Networks in HPV-Induced Carcinogenesis
Source: mSphere. 2019 Mar 27;4(2):e00129-19. doi: 10.1128/mSphere.00129-19 (PMC6437273; doi:10.1128/mSphere.00129-19)

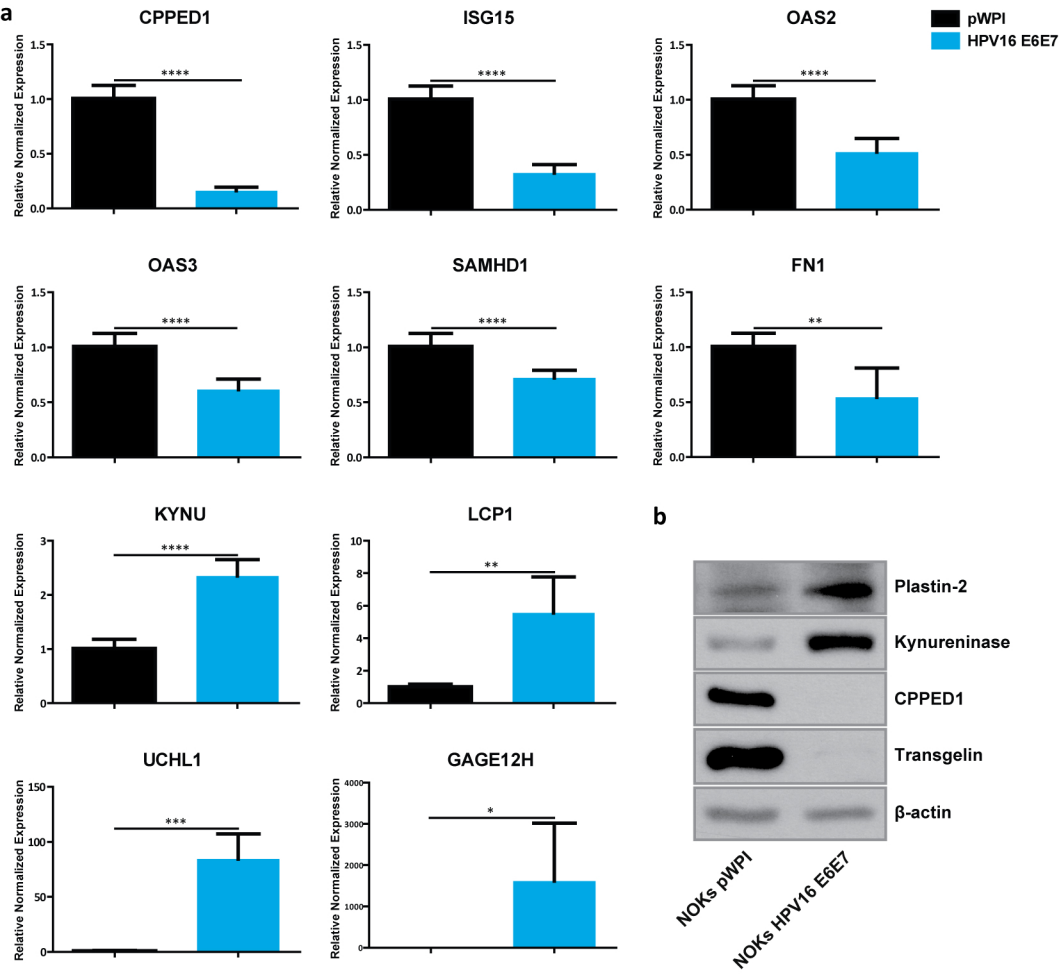

Supplement: FIG S1 [file mSphere.00129-19-sf001.pdf]

**BCL2L1**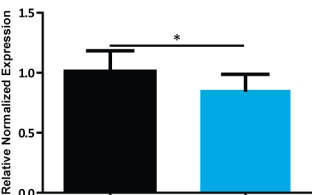**CCND1**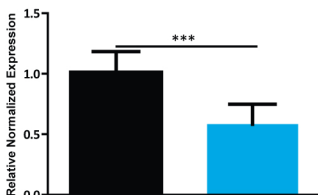**CDC25B**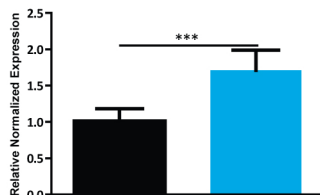**CLDN7**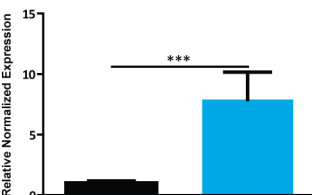**COL5A1**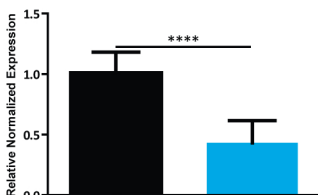**HERC6**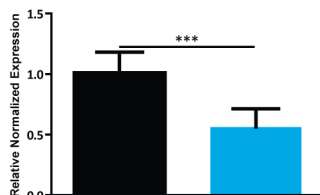**KYNU1**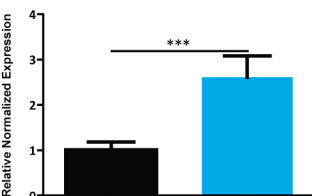**KYNU2**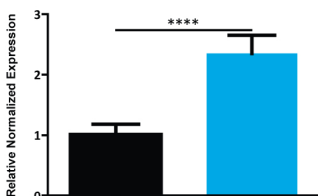**LCP1**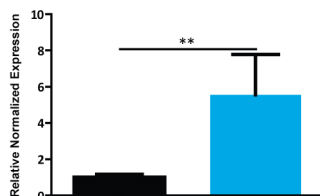**MMP2**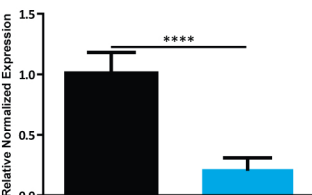**PLSCR1**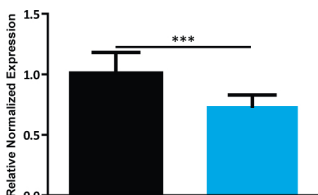**SERPINE1**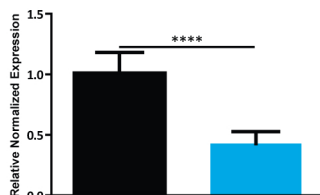**SPDEF**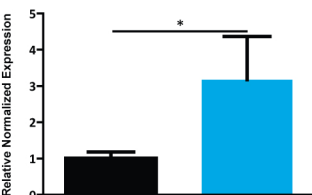**TGM2**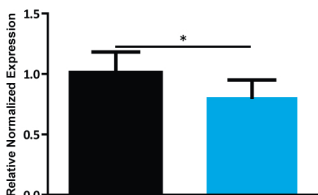

■ pWPI  
■ HPV16 E6E7

Supplement: FIG S2 [file mSphere.00129-19-sf002.pdf]
