# Supplementary material for: Combined Transcriptome and Proteome Analysis of Immortalized Human Keratinocytes Expressing Human Papillomavirus 16 (HPV16) Oncogenes Reveals Novel Key Factors and Networks in HPV-Induced Carcinogenesis
Source: mSphere. 2019 Mar 27;4(2):e00129-19. doi: 10.1128/mSphere.00129-19 (PMC6437273; doi:10.1128/mSphere.00129-19)
Supplement: TABLE S1 [file mSphere.00129-19-st001.docx]

**Table S1**

| **Primer name** | **Sequence 5’ 🡪 3’** | **application** |
| --- | --- | --- |
| P2A-pWPI-F1 | CAGGCGCGCCGGAAGCGGAGCTACTAACTTCAGCCTGCTG | cloning |
| P2A-pWPI-R1 | TGCTTAACGCGTAGGTCCAGGGTTCTCCTCCAC | cloning |
| P2A-pWPI-F2 | TAAGCACCTGCAGGTAAGCAGGCGCGCCGGAAGCGGA | cloning |
| P2A-pWPI-R2 | TGCTTAACTAGTTGCTTAACGCGTAGGTCCAGGGT | cloning |
| 16E6 pCMV-F | TAAGCACTGCAGATGTTTCAGGACCCACAGGA | cloning |
| 16E6 pCMV-R | TGCTTAGTCGACTTACAGCTGGGTTTCTCTACG | cloning |
| 3Flag-P2A-pWPI-F | TAAGCAACGCGTGATTACAAGGATGACGACGAT | cloning |
| 16E6-3Flag-P2A-pWPI-R | TGCTTAACTAGTTTACAGCTGGGTTTCTCTACGTGT | cloning |
| 16E7-Strep-P2A-pWPI-F | TAAGCACCTGCAGGGCCACCATGCATGGAGATACACCTACA | cloning |
| 16E7-Strep-P2A-pWPI-R1 | CTCGAACTGCGGGTGGCTCCATGGTTTCTGAGAACAGATGGG | cloning |
| Strep-P2A-pWPI-R2 | ACCTCCGGAACCTCCACCTTTCTCGAACTGCGGGTGGCTCCA | cloning |
| Strep-P2A-pWPI-R3 | CCACGATCCACCTCCCGATCCACCTCCGGAACCTCCACCTTT | cloning |
| Strep-P2A-pWPI-R4 | TTTTTCGAACTGCGGGTGGCTCCACGATCCACCTCCCGATCC | cloning |
| Strep-P2A-pWPI-R5 | TGCTTAGGCGCGCCGTTTTTCGAACTGCGGGTGGCT | cloning |
| ACTB F | CATCCGCAAAGACCTGTACG | RT-PCR |
| ACTB R | CCTGCTTGCTGATCCACATC | RT-PCR |
| HPV16E6 F | ACTGCAATGTTTCAGGACCC | RT-PCR |
| HPV16E6 R | TCAGGACACAGTGGCTTTTG | RT-PCR |
| HPV16-E6 F | TCAGGACCCACAGGAGCG | qPCR |
| HPV16-E6 R | CCTCACGTCGCAGTAACTGTTG | qPCR |
| HPV16E7 F | CAGCTCAGAGGAGGAGGATG | RT-PCR |
| HPV16E7 R | GCCCATTAACAGGTCTTCCA | RT-PCR |
| TOP1 F | GAGCTGAGCCAGTTGTCCTA | qPCR |
| TOP1 R | TTTGCCTGGTAGAACGCTGA | qPCR |
| CPPED1 F | GGTCACGATTCCCAAAACGC | qPCR |
| CPPED1 R | GTGCCTACTTGGCTCCTTGT | qPCR |
| ISG15 F | GGTGGACAAATGCGACGAAC | qPCR |
| ISG15 R | TCGAAGGTCAGCCAGAACAG | qPCR |
| OAS2 F | GCCAACGTGACATCCTCGAT | qPCR |
| OAS2 R | GCCAGCACCTCGAAAGAGAT | qPCR |
| OAS3 F | AGGCCCAGCTCTCAAGTCTA | qPCR |
| OAS3 R | GGCTCTTCAGCTTGGTAGGG | qPCR |
| SAMHD1 F | TTTGCCCGTGTCTGTGAAGT | qPCR |
| SAMHD1 R | TGCGAGTGTGGAACATGTCA | qPCR |
| FN1 F | AGCCGAGGTTTTAACTGCGA | qPCR |
| FN1 R | CCCACTCGGTAAGTGTTCCC | qPCR |
| KYNU F | AGCAAGCGACAATGAAGGCA | qPCR |
| KYNU R | TTATTGTTAGCTGGCACCCCC | qPCR |
| LCP1 F | AGGCTTGACAAAGCTGTTCTG | qPCR |
| LCP1 R | ACACTGATCCTCTGGCCATTTT | qPCR |
| UCHL1 F | AAGGCCAATGTCGGGTAGATG | qPCR |
| UCHL1 R | GACTTCTCCTTGCTCACGCT | qPCR |
| GAGE12H F | CCTTCGCCCACGTGAAGA | qPCR |
| GAGE12H R | GAAAAAGAGTCCGGACGGCA | qPCR |
| BCL2L1 F | GATCCCCATGGCAGCAGTAA | qPCR |
| BCL2L1 R | GTGATGTGGAGCTGGGATGT | qPCR |
| CCND1 F | CAGATCATCCGCAAACACGC | qPCR |
| CCND1 R | AAGTTGTTGGGGCTCCTCAG | qPCR |
| CDC25B F | CTCCTGCTGGGATCTCATGG | qPCR |
| CDC25B R | CATGGTCTGGGTGAGGGTG | qPCR |
| CLDN7 F | CCTAATGGTGGTCTCCCTGG | qPCR |
| CLDN7 R | TGGCTATACGGGCCTTCTTC | qPCR |
| COL5A1 F | TACTACACGCCCTCACCGTA | qPCR |
| COL5A1 R | TCCAAGTCATCCGCACCTTC | qPCR |
| HERC6 F | TTTTCGTGGGGAAAGAACAGC | qPCR |
| HERC6 R | TCCCCAGCCAAACGAAGTC | qPCR |
| MMP2 F | TCCCCTTCTTGTTCAATGGCA | qPCR |
| MMP2 R | AGCCGTACTTGCCATCCTTC | qPCR |
| PLSCR1 F | GGCACCCATGTCTACCAAAGT | qPCR |
| PLSCR1 R | CACAACAGCTGCACACAACA | qPCR |
| SERPINE1 F | AGAGCGCTGTCAAGAAGACC | qPCR |
| SERPINE1 R | AGTTCTCAGAGGTGCCTTGC | qPCR |
| SPDEF F | CACAGACCCAGTCCTCCAAG | qPCR |
| SPDEF R | CAGAGGCAGCACTCAGGTTG | qPCR |
| TGM2 F | ATCGGCCTGTATCGCCTCA | qPCR |
| TGM2 R | CAGGCGTTGAAGAGCAAAATGA | qPCR |
