# Supplementary material for: Combined Transcriptome and Proteome Analysis of Immortalized Human Keratinocytes Expressing Human Papillomavirus 16 (HPV16) Oncogenes Reveals Novel Key Factors and Networks in HPV-Induced Carcinogenesis
Source: mSphere. 2019 Mar 27;4(2):e00129-19. doi: 10.1128/mSphere.00129-19 (PMC6437273; doi:10.1128/mSphere.00129-19)
Supplement: TABLE S2 [file mSphere.00129-19-st002.docx]

**Table S2**

|  | **Human genome** | | | | | | | |
| --- | --- | --- | --- | --- | --- | --- | --- | --- |
| **Sample Name** | **Reads in total** | **overall alignment rate** | **aligned 0times** | **aligned 0times (rate)** | **aligned 1 time** | **aligned 1 time (rate)** | **align >1 times** | **align >1 times (rate)** |
| NOKs pWPI 1 | 31717899 | 96.23% | 1196624 | 3.77% | 26713795 | 84.22% | 3807480 | 12.00% |
| NOKs pWPI 2 | 39108231 | 96.14% | 1508875 | 3.86% | 32748659 | 83.74% | 4850697 | 12.40% |
| NOKs pWPI 3 | 32961787 | 96.19% | 1257320 | 3.81% | 27816439 | 84.39% | 3888028 | 11.80% |
|  |  |  |  |  |  |  |  |  |
| NOKs HPV16E6E7 1 | 34249638 | 96.27% | 1277837 | 3.73% | 28710857 | 83.83% | 4260944 | 12.44% |
| NOKs HPV16E6E7 2 | 37173393 | 96.37% | 1349261 | 3.63% | 31260057 | 84.09% | 4564075 | 12.28% |
| NOKs HPV16E6E7 3 | 40061967 | 96.35% | 1461575 | 3.65% | 33786451 | 84.34% | 4813941 | 12.02% |
|  | **HPV16 genome** | | | | | | | |
| **Sample Name** | **Reads in total** | **overall alignment rate** | **aligned 0times (rate)** | **aligned 0times (rate)** | **aligned 1 time** | **aligned 1 time (rate)** | **align >1 times** | **align >1 times (rate)** |
| NOKs pWPI 1 | 1195023 | 0.00% | 1195022 | 100.00% | 1 | 0.00% | 0 | 0.00% |
| NOKs pWPI 2 | 1505431 | 0.00% | 1505428 | 100.00% | 3 | 0.00% | 0 | 0.00% |
| NOKs pWPI 3 | 1256688 | 0.00% | 1256686 | 100.00% | 2 | 0.00% | 0 | 0.00% |
|  |  |  |  |  |  |  |  |  |
| NOKs HPV16E6E7 1 | 1277628 | 2.57% | 1244826 | 97.43% | 32802 | 2.57% | 0 | 0.00% |
| NOKs HPV16E6E7 2 | 1348985 | 2.76% | 1311691 | 97.24% | 37294 | 2.76% | 0 | 0.00% |
| NOKs HPV16E6E7 3 | 1461312 | 2.46% | 1425435 | 97.54% | 35877 | 2.46% | 0 | 0.00% |
