# Supplementary material for: Combined Transcriptome and Proteome Analysis of Immortalized Human Keratinocytes Expressing Human Papillomavirus 16 (HPV16) Oncogenes Reveals Novel Key Factors and Networks in HPV-Induced Carcinogenesis
Source: mSphere. 2019 Mar 27;4(2):e00129-19. doi: 10.1128/mSphere.00129-19 (PMC6437273; doi:10.1128/mSphere.00129-19)
Supplement: TABLE S3 [file mSphere.00129-19-st003.docx]

**Table S3**

| **Gene ID** | **Gene name** | **SILAC** | **Average RNA-seq** | **BtDe** | **HsStDe** | **SmDe** | **SmSu** |
| --- | --- | --- | --- | --- | --- | --- | --- |
| ENSG00000154277 | UCHL1 | 2.598 | 5.582 | 2.810 | 7.051 | 6.284 | 6.183 |
| ENSG00000136167 | LCP1 | 2.209 | 2.282 | 1.436 | 2.620 | 2.611 | 2.460 |
| ENSG00000143320 | CRABP2 | 1.625 | 1.894 | 1.600 | 2.047 | 1.934 | 1.996 |
| ENSG00000178726 | THBD | 1.327 | 1.659 | 1.450 | 1.762 | 1.708 | 1.717 |
| ENSG00000166165 | CKB | 1.250 | 0.903 | 0.853 | 0.841 | 0.972 | 0.947 |
| ENSG00000081181 | ARG2 | 1.249 | 0.840 | 0.524 | 0.864 | 1.053 | 0.918 |
| ENSG00000261150 | EPPK1 | 1.041 | 1.727 | N/A | 1.176 | 1.963 | 2.043 |
| ENSG00000176890 | TYMS | 0.989 | 0.273 | N/A | 0.264 | 0.282 | N/A |
| ENSG00000113522 | RAD50 | 0.950 | 0.842 | N/A | 0.720 | 0.855 | 0.951 |
| ENSG00000115919 | KYNU | 0.803 | 1.008 | N/A | 1.011 | 1.041 | 0.972 |
| ENSG00000151689 | INPP1 | 0.771 | 0.863 | N/A | 0.917 | 0.855 | 0.817 |
| ENSG00000271303 | SRXN1 | 0.768 | 0.423 | N/A | 0.448 | 0.398 | N/A |
| ENSG00000146648 | EGFR | 0.710 | 0.492 | N/A | 0.434 | 0.549 | N/A |
| ENSG00000058673 | ZC3H11A | 0.686 | 0.810 | N/A | 1.166 | 0.340 | 0.925 |
| ENSG00000132646 | PCNA | 0.673 | 0.481 | 0.503 | 0.462 | 0.479 | N/A |
| ENSG00000163344 | PMVK | 0.654 | 0.557 | 0.492 | 0.612 | 0.551 | 0.572 |
| ENSG00000125877 | ITPA | 0.628 | 0.550 | 0.534 | 0.557 | 0.560 | N/A |
| ENSG00000173083 | HPSE | 0.628 | 0.466 | N/A | 0.426 | 0.506 | N/A |
| ENSG00000101343 | CRNKL1 | 0.598 | 0.624 | N/A | 0.696 | 0.552 | N/A |
| ENSG00000183696 | UPP1 | 0.598 | 0.702 | 0.713 | 0.709 | 0.704 | 0.679 |
| ENSG00000151729 | SLC25A4 | 0.592 | 0.583 | N/A | 0.607 | 0.559 | N/A |
| ENSG00000089048 | ESF1 | 0.591 | 0.579 | N/A | 0.608 | 0.550 | N/A |
| ENSG00000135842 | FAM129A | 0.534 | 0.803 | N/A | 0.817 | 0.789 | N/A |
| ENSG00000070814 | TCOF1 | 0.533 | 0.650 | 0.653 | 0.717 | 0.581 | N/A |
| ENSG00000117748 | RPA2 | 0.530 | 0.427 | N/A | 0.353 | 0.501 | N/A |
| ENSG00000183963 | SMTN | 0.526 | 0.577 | 0.504 | N/A | 0.535 | 0.694 |
| ENSG00000073282 | TP63 | 0.519 | 0.895 | N/A | 0.953 | 0.639 | 1.093 |
| ENSG00000161011 | SQSTM1 | 0.497 | 0.611 | 0.560 | 0.641 | 0.592 | 0.649 |
| ENSG00000088833 | NSFL1C | 0.488 | 0.640 | 0.566 | 0.668 | 0.642 | 0.683 |
| ENSG00000130305 | NSUN5 | 0.474 | 0.540 | N/A | 0.610 | 0.469 | N/A |
| ENSG00000168404 | MLKL | 0.474 | 0.400 | N/A | 0.330 | 0.470 | N/A |
| ENSG00000140263 | SORD | 0.454 | 0.761 | N/A | 0.711 | 0.811 | N/A |
| ENSG00000145220 | LYAR | 0.451 | 0.513 | 0.406 | 0.620 | N/A | N/A |
| ENSG00000125818 | PSMF1 | 0.448 | 0.539 | 0.486 | 0.513 | 0.666 | 0.492 |
| ENSG00000088930 | XRN2 | 0.444 | 0.485 | 0.464 | 0.527 | 0.464 | N/A |
| ENSG00000183010 | PYCR1 | 0.444 | 0.773 | 0.689 | 0.818 | 0.836 | 0.750 |
| ENSG00000137309 | HMGA1 | 0.431 | 0.825 | 0.735 | 0.911 | 0.828 | N/A |
| ENSG00000071539 | TRIP13 | 0.429 | 0.378 | N/A | 0.367 | 0.389 | N/A |
| ENSG00000178999 | AURKB | 0.427 | 0.515 | 0.464 | 0.616 | 0.463 | N/A |
| ENSG00000164916 | FOXK1 | 0.416 | 0.674 | N/A | 0.671 | 0.796 | 0.557 |
| ENSG00000089195 | TRMT6 | 0.413 | 0.347 | 0.532 | N/A | -0.324 | 0.834 |
| ENSG00000247077 | PGAM5 | 0.402 | 0.504 | 0.458 | N/A | 0.520 | 0.533 |
| ENSG00000108106 | UBE2S | 0.394 | 0.544 | 0.529 | N/A | 0.559 | N/A |
| ENSG00000121552 | CSTA | 0.391 | -0.459 | N/A | -0.439 | -0.480 | N/A |
| ENSG00000126803 | HSPA2 | 0.386 | 1.266 | N/A | 1.214 | 1.429 | 1.153 |
| ENSG00000124164 | VAPB | -0.379 | -0.497 | N/A | -0.397 | -0.328 | -0.767 |
| ENSG00000101608 | MYL12A | -0.386 | -0.325 | N/A | -0.258 | -0.393 | N/A |
| ENSG00000117519 | CNN3 | -0.387 | -0.417 | -0.359 | -0.427 | -0.464 | N/A |
| ENSG00000164111 | ANXA5 | -0.390 | -0.324 | -0.318 | N/A | -0.330 | N/A |
| ENSG00000121060 | TRIM25 | -0.399 | -0.408 | N/A | -0.411 | -0.391 | -0.421 |
| ENSG00000172936 | MYD88 | -0.407 | -0.685 | -0.585 | N/A | -0.652 | -0.818 |
| ENSG00000129353 | SLC44A2 | -0.415 | -0.521 | -0.397 | N/A | -0.505 | -0.661 |
| ENSG00000124942 | AHNAK | -0.415 | -0.504 | -0.459 | -0.501 | -0.552 | N/A |
| ENSG00000156675 | RAB11FIP1 | -0.418 | -0.797 | -0.554 | -0.704 | -0.652 | -1.280 |
| ENSG00000168615 | ADAM9 | -0.425 | -0.491 | -0.468 | -0.431 | -0.518 | -0.547 |
| ENSG00000137364 | TPMT | -0.429 | -0.622 | N/A | -0.622 | -0.622 | N/A |
| ENSG00000178209 | PLEC | -0.434 | -0.493 | -0.423 | -0.470 | -0.585 | N/A |
| ENSG00000138772 | ANXA3 | -0.436 | -0.474 | -0.478 | -0.509 | -0.436 | N/A |
| ENSG00000196141 | SPATS2L | -0.438 | -0.340 | N/A | -0.439 | -0.242 | N/A |
| ENSG00000150093 | ITGB1 | -0.442 | -0.468 | -0.472 | -0.518 | -0.414 | N/A |
| ENSG00000065154 | OAT | -0.447 | -0.470 | -0.439 | -0.442 | -0.529 | N/A |
| ENSG00000138413 | IDH1 | -0.449 | -0.493 | -0.465 | -0.577 | -0.436 | N/A |
| ENSG00000119655 | NPC2 | -0.459 | -0.442 | -0.396 | -0.529 | -0.401 | N/A |
| ENSG00000205542 | TMSB4X | -0.462 | -0.630 | -0.442 | -0.820 | -0.628 | N/A |
| ENSG00000002549 | LAP3 | -0.464 | -0.347 | N/A | -0.322 | -0.372 | N/A |
| ENSG00000221926 | TRIM16 | -0.464 | -0.574 | N/A | -0.738 | -0.410 | N/A |
| ENSG00000064601 | CTSA | -0.465 | -0.604 | -0.531 | -0.622 | -0.500 | -0.763 |
| ENSG00000182718 | ANXA2 | -0.468 | -0.419 | -0.362 | -0.468 | -0.425 | N/A |
| ENSG00000101367 | MAPRE1 | -0.485 | -0.297 | N/A | -0.266 | -0.328 | N/A |
| ENSG00000105971 | CAV2 | -0.489 | -0.298 | N/A | -0.322 | -0.274 | N/A |
| ENSG00000101346 | POFUT1 | -0.490 | -0.391 | N/A | -0.355 | -0.401 | -0.415 |
| ENSG00000055332 | EIF2AK2 | -0.499 | -0.693 | N/A | -0.630 | -0.472 | -0.977 |
| ENSG00000041353 | RAB27B | -0.501 | -0.649 | N/A | -0.815 | -0.482 | N/A |
| ENSG00000163191 | S100A11 | -0.505 | -0.341 | -0.313 | -0.324 | -0.386 | N/A |
| ENSG00000010278 | CD9 | -0.506 | -0.299 | N/A | -0.300 | -0.298 | N/A |
| ENSG00000107201 | DDX58 | -0.508 | -0.800 | N/A | -0.800 | -0.800 | N/A |
| ENSG00000101152 | DNAJC5 | -0.511 | -0.323 | N/A | -0.326 | -0.320 | N/A |
| ENSG00000100003 | SEC14L2 | -0.520 | -0.814 | -0.617 | -0.790 | -0.656 | -1.192 |
| ENSG00000213463 | SYNJ2BP | -0.531 | -0.482 | N/A | -0.549 | -0.414 | N/A |
| ENSG00000169504 | CLIC4 | -0.533 | -0.724 | -0.647 | -0.801 | N/A | N/A |
| ENSG00000122786 | CALD1 | -0.541 | -0.357 | N/A | -0.417 | -0.297 | N/A |
| ENSG00000166913 | YWHAB | -0.545 | -0.659 | -0.516 | -1.030 | -0.432 | N/A |
| ENSG00000122359 | ANXA11 | -0.550 | -0.341 | N/A | -0.333 | -0.350 | N/A |
| ENSG00000182795 | C1orf116 | -0.551 | -0.590 | -0.457 | -0.526 | -0.528 | -0.848 |
| ENSG00000105974 | CAV1 | -0.556 | -0.510 | -0.531 | -0.597 | -0.400 | N/A |
| ENSG00000197747 | S100A10 | -0.559 | -0.456 | -0.431 | -0.436 | -0.440 | -0.517 |
| ENSG00000196937 | FAM3C | -0.564 | -0.707 | -0.677 | -0.718 | -0.471 | -0.962 |
| ENSG00000115504 | EHBP1 | -0.568 | -0.564 | N/A | -0.516 | -0.485 | -0.692 |
| ENSG00000136153 | LMO7 | -0.568 | -0.729 | N/A | -0.527 | -0.646 | -1.013 |
| ENSG00000197321 | SVIL | -0.568 | -0.597 | N/A | -0.575 | -0.631 | -0.585 |
| ENSG00000189221 | MAOA | -0.573 | -0.563 | N/A | -0.458 | -0.401 | -0.830 |
| ENSG00000109452 | INPP4B | -0.583 | -0.414 | N/A | -0.479 | -0.349 | N/A |
| ENSG00000180185 | FAHD1 | -0.585 | -0.690 | N/A | -0.916 | -0.579 | -0.577 |
| ENSG00000189060 | H1F0 | -0.600 | -0.462 | -0.420 | -0.450 | -0.499 | -0.477 |
| ENSG00000277443 | MARCKS | -0.618 | -0.600 | -0.558 | -0.642 | N/A | N/A |
| ENSG00000188313 | PLSCR1 | -0.642 | -0.635 | -0.567 | -0.553 | -0.785 | N/A |
| ENSG00000175866 | BAIAP2 | -0.666 | -0.424 | N/A | -0.392 | -0.456 | N/A |
| ENSG00000068079 | IFI35 | -0.670 | -0.725 | -0.663 | -0.701 | -0.811 | N/A |
| ENSG00000138119 | MYOF | -0.682 | -0.649 | -0.582 | -0.632 | -0.629 | -0.753 |
| ENSG00000184254 | ALDH1A3 | -0.690 | -0.803 | -0.784 | -0.814 | -0.659 | -0.954 |
| ENSG00000053747 | LAMA3 | -0.694 | -0.563 | -0.588 | -0.385 | -0.715 | N/A |
| ENSG00000125730 | C3 | -0.696 | -0.841 | -0.597 | -0.735 | -0.826 | -1.207 |
| ENSG00000214078 | CPNE1 | -0.710 | -0.509 | -0.451 | -0.420 | -0.541 | -0.625 |
| ENSG00000124217 | MOCS3 | -0.768 | -0.464 | N/A | -0.445 | -0.484 | N/A |
| ENSG00000013588 | GPRC5A | -0.793 | -0.868 | -0.864 | -0.660 | -0.948 | -1.001 |
| ENSG00000159348 | CYB5R1 | -0.796 | -0.830 | -0.776 | -0.761 | -0.792 | -0.992 |
| ENSG00000148671 | ADIRF | -0.798 | -0.957 | -0.918 | -1.008 | -0.868 | -1.032 |
| ENSG00000173193 | PARP14 | -0.824 | -0.707 | -0.603 | -0.643 | -0.665 | -0.916 |
| ENSG00000185567 | AHNAK2 | -0.835 | -1.209 | -0.811 | -1.209 | -1.359 | -1.456 |
| ENSG00000117984 | CTSD | -0.856 | -0.671 | -0.527 | -0.624 | -0.714 | -0.821 |
| ENSG00000148180 | GSN | -0.864 | -0.512 | N/A | -0.483 | -0.542 | N/A |
| ENSG00000105755 | ETHE1 | -0.880 | -0.591 | -0.537 | -0.569 | -0.624 | -0.636 |
| ENSG00000135821 | GLUL | -0.886 | -0.920 | -0.770 | -0.757 | -0.766 | -1.387 |
| ENSG00000130589 | HELZ2 | -0.895 | -0.914 | -0.689 | -0.886 | -1.010 | -1.073 |
| ENSG00000151726 | ACSL1 | -0.962 | -0.759 | -0.605 | -0.809 | -0.664 | -0.961 |
| ENSG00000101347 | SAMHD1 | -0.970 | -1.113 | -0.963 | -1.156 | -1.139 | -1.192 |
| ENSG00000111331 | OAS3 | -0.979 | -0.830 | -0.761 | -0.871 | -0.755 | -0.932 |
| ENSG00000092964 | DPYSL2 | -0.996 | -0.905 | -0.633 | -0.955 | -0.949 | -1.085 |
| ENSG00000166741 | NNMT | -1.009 | -1.151 | -0.850 | -1.140 | -1.219 | -1.398 |
| ENSG00000115415 | STAT1 | -1.010 | -1.401 | -1.182 | -1.186 | -1.466 | -1.771 |
| ENSG00000205413 | SAMD9 | -1.036 | -1.375 | -1.081 | -1.426 | -1.463 | -1.529 |
| ENSG00000175662 | TOM1L2 | -1.038 | -0.867 | -0.683 | N/A | -0.863 | -1.054 |
| ENSG00000108679 | LGALS3BP | -1.048 | -0.901 | -0.751 | -0.768 | -0.828 | -1.256 |
| ENSG00000198959 | TGM2 | -1.089 | -1.190 | N/A | -1.223 | -0.913 | -1.434 |
| ENSG00000166535 | A2ML1 | -1.119 | -1.637 | -1.279 | -1.809 | -1.658 | -1.805 |
| ENSG00000137628 | DDX60 | -1.193 | -1.797 | -1.192 | -2.031 | -1.999 | -1.966 |
| ENSG00000117228 | GBP1 | -1.204 | -1.045 | -0.691 | -1.064 | -1.350 | -1.076 |
| ENSG00000163840 | DTX3L | -1.481 | -0.619 | N/A | -0.640 | -0.538 | -0.680 |
| ENSG00000111335 | OAS2 | -1.539 | -1.382 | -1.134 | -1.427 | -1.432 | -1.534 |
| ENSG00000155850 | SLC26A2 | -1.544 | -1.656 | -1.277 | -1.813 | -1.710 | -1.824 |
| ENSG00000115414 | FN1 | -1.575 | -1.825 | -1.686 | -1.724 | -1.791 | -2.101 |
| ENSG00000187608 | ISG15 | -1.599 | -1.587 | -1.433 | -1.615 | -1.664 | -1.638 |
| ENSG00000157601 | MX1 | -2.075 | -1.826 | -1.391 | -1.767 | -1.973 | -2.175 |
| ENSG00000149591 | TAGLN | -2.090 | -1.962 | -1.194 | -2.281 | -2.166 | -2.206 |
| ENSG00000103381 | CPPED1 | -2.657 | -2.872 | -1.546 | -3.496 | -3.115 | -3.331 |
| ENSG00000134283 | PPHLN1 | 1.517 | 0.040 | -0.063 | 0.085 | -0.102 | 0.240 |
| ENSG00000070669 | ASNS | 0.955 | 0.263 | 0.148 | 0.175 | 0.138 | 0.591 |
| ENSG00000151465 | CDC123 | 0.911 | 0.112 | 0.096 | -0.039 | 0.015 | 0.376 |
| ENSG00000124610 | HIST1H1A | 0.811 | -1.011 | N/A | -0.991 | -1.031 | N/A |
| ENSG00000164070 | HSPA4L | 0.802 | 0.558 | 0.311 | 0.412 | 0.517 | 0.991 |
| ENSG00000072274 | TFRC | 0.750 | 0.438 | 0.321 | 0.350 | 0.401 | 0.679 |
| ENSG00000115008 | IL1A | 0.741 | 0.196 | 0.160 | 0.217 | 0.161 | 0.245 |
| ENSG00000103152 | MPG | 0.739 | 0.176 | 0.106 | 0.188 | 0.151 | 0.261 |
| ENSG00000172922 | RNASEH2C | 0.672 | 0.202 | 0.157 | 0.078 | 0.287 | 0.288 |
| ENSG00000112159 | MDN1 | 0.671 | 0.016 | -0.002 | 0.045 | -0.143 | 0.166 |
| ENSG00000156970 | BUB1B | 0.659 | 0.362 | 0.150 | 0.212 | 0.327 | 0.762 |
| ENSG00000168724 | DNAJC21 | 0.625 | 0.127 | 0.115 | 0.188 | 0.162 | 0.041 |
| ENSG00000095059 | DHPS | 0.622 | 0.471 | 0.225 | 0.301 | 0.114 | 1.243 |
| ENSG00000137337 | MDC1 | 0.600 | 0.480 | 0.413 | N/A | 0.473 | 0.555 |
| ENSG00000197498 | RPF2 | 0.593 | 0.219 | -0.018 | 0.083 | -0.097 | 0.908 |
| ENSG00000241794 | SPRR2A | 0.589 | 0.344 | 0.226 | 0.345 | 0.383 | 0.422 |
| ENSG00000185238 | PRMT3 | 0.569 | 0.550 | 0.240 | 0.335 | 0.511 | 1.115 |
| ENSG00000167196 | FBXO22 | 0.558 | 0.164 | 0.089 | 0.248 | 0.206 | 0.113 |
| ENSG00000153827 | TRIP12 | 0.556 | 0.057 | -0.010 | -0.087 | 0.046 | 0.280 |
| ENSG00000196712 | NF1 | 0.537 | -0.281 | -0.003 | -0.066 | -0.185 | -0.871 |
| ENSG00000147592 | LACTB2 | 0.530 | 0.471 | 0.359 | 0.664 | 0.405 | 0.455 |
| ENSG00000149948 | HMGA2 | 0.528 | 0.571 | 0.239 | 0.657 | 0.705 | 0.685 |
| ENSG00000123178 | SPRYD7 | 0.514 | -0.168 | -0.076 | -0.062 | -0.304 | -0.228 |
| ENSG00000092148 | HECTD1 | 0.513 | 0.031 | -0.031 | -0.023 | 0.146 | 0.032 |
| ENSG00000183386 | FHL3 | 0.511 | 0.624 | 0.472 | 0.646 | 0.645 | 0.733 |
| ENSG00000020922 | MRE11A | 0.508 | -0.128 | 0.018 | -0.073 | 0.135 | -0.594 |
| ENSG00000148688 | RPP30 | 0.495 | 0.020 | 0.022 | 0.085 | -0.015 | -0.011 |
| ENSG00000106399 | RPA3 | 0.488 | 0.163 | 0.068 | 0.161 | 0.075 | 0.349 |
| ENSG00000198604 | BAZ1A | 0.486 | 0.213 | 0.146 | 0.169 | 0.098 | 0.438 |
| ENSG00000127554 | GFER | 0.481 | 0.360 | 0.122 | 0.274 | 0.519 | 0.526 |
| ENSG00000122965 | RBM19 | 0.478 | 0.336 | 0.282 | 0.397 | 0.240 | 0.424 |
| ENSG00000182481 | KPNA2 | 0.478 | 0.074 | 0.091 | -0.019 | -0.108 | 0.332 |
| ENSG00000145041 | VPRBP | 0.477 | 0.354 | 0.215 | 0.404 | 0.387 | 0.410 |
| ENSG00000011260 | UTP18 | 0.477 | 0.053 | 0.165 | 0.121 | -0.153 | 0.079 |
| ENSG00000104889 | RNASEH2A | 0.472 | 0.225 | 0.140 | 0.147 | 0.132 | 0.481 |
| ENSG00000132603 | NIP7 | 0.460 | -0.038 | 0.091 | 0.079 | 0.142 | -0.465 |
| ENSG00000198522 | GPN1 | 0.458 | -0.147 | 0.036 | 0.000 | 0.024 | -0.648 |
| ENSG00000186010 | NDUFA13 | 0.454 | 0.093 | 0.147 | 0.181 | 0.160 | -0.115 |
| ENSG00000055609 | KMT2C | 0.444 | -0.102 | -0.030 | 0.031 | -0.130 | -0.281 |
| ENSG00000066583 | ISOC1 | 0.440 | 0.666 | 0.257 | 0.301 | 0.609 | 1.497 |
| ENSG00000135521 | LTV1 | 0.437 | 0.060 | 0.057 | 0.110 | 0.016 | 0.057 |
| ENSG00000026103 | FAS | 0.434 | 0.223 | 0.088 | 0.296 | 0.133 | 0.373 |
| ENSG00000132383 | RPA1 | 0.433 | 0.173 | 0.121 | 0.050 | 0.108 | 0.414 |
| ENSG00000187522 | HSPA14 | 0.433 | 0.134 | 0.019 | 0.062 | 0.075 | 0.378 |
| ENSG00000105676 | ARMC6 | 0.428 | 0.424 | 0.403 | 0.379 | 0.416 | 0.497 |
| ENSG00000149554 | CHEK1 | 0.426 | 0.406 | 0.231 | 0.435 | 0.317 | 0.641 |
| ENSG00000150593 | PDCD4 | 0.425 | 0.128 | 0.105 | 0.196 | 0.138 | 0.073 |
| ENSG00000136045 | PWP1 | 0.425 | 0.349 | -0.065 | -0.049 | 0.144 | 1.367 |
| ENSG00000166788 | SAAL1 | 0.423 | 0.133 | 0.141 | 0.202 | 0.157 | 0.031 |
| ENSG00000077514 | POLD3 | 0.421 | 0.104 | 0.010 | 0.228 | 0.010 | 0.169 |
| ENSG00000035928 | RFC1 | 0.417 | -0.020 | 0.056 | -0.005 | 0.002 | -0.134 |
| ENSG00000141076 | CIRH1A | 0.413 | 0.161 | 0.145 | 0.140 | 0.017 | 0.341 |
| ENSG00000116679 | IVNS1ABP | 0.409 | -0.245 | -0.176 | -0.249 | -0.248 | -0.308 |
| ENSG00000185088 | RPS27L | 0.405 | -0.150 | -0.093 | -0.115 | -0.173 | -0.220 |
| ENSG00000198824 | CHAMP1 | 0.400 | -0.185 | 0.071 | 0.121 | -0.011 | -0.920 |
| ENSG00000134057 | CCNB1 | 0.400 | 0.187 | 0.141 | 0.229 | 0.084 | 0.295 |
| ENSG00000133731 | IMPA1 | 0.400 | 0.181 | 0.059 | 0.308 | 0.215 | 0.143 |
| ENSG00000175376 | EIF1AD | 0.396 | 0.237 | 0.133 | 0.249 | 0.263 | 0.304 |
| ENSG00000187164 | KIAA1598 | 0.396 | -0.288 | 0.041 | 0.162 | -0.311 | -1.044 |
| ENSG00000234127 | TRIM26 | 0.392 | 0.196 | 0.205 | N/A | 0.337 | 0.046 |
| ENSG00000125484 | GTF3C4 | 0.391 | 0.319 | 0.065 | 0.288 | 0.088 | 0.836 |
| ENSG00000136146 | MED4 | 0.391 | -0.028 | -0.141 | -0.187 | -0.016 | 0.233 |
| ENSG00000139746 | RBM26 | 0.390 | 0.200 | 0.040 | 0.049 | 0.138 | 0.574 |
| ENSG00000078237 | TIGAR | 0.389 | 0.248 | 0.175 | 0.258 | 0.196 | 0.363 |
| ENSG00000119421 | NDUFA8 | 0.388 | -0.002 | 0.016 | 0.016 | -0.033 | -0.008 |
| ENSG00000134371 | CDC73 | 0.387 | -0.103 | -0.003 | -0.074 | -0.144 | -0.191 |
| ENSG00000063854 | HAGH | -0.380 | 0.107 | 0.051 | 0.128 | 0.087 | 0.162 |
| ENSG00000047932 | GOPC | -0.382 | -0.347 | -0.301 | -0.385 | -0.280 | -0.422 |
| ENSG00000084207 | GSTP1 | -0.382 | -0.008 | 0.028 | -0.101 | 0.015 | 0.024 |
| ENSG00000176788 | BASP1 | -0.383 | -0.302 | -0.228 | -0.477 | -0.184 | -0.318 |
| ENSG00000137106 | GRHPR | -0.385 | -0.292 | -0.185 | -0.340 | -0.256 | -0.387 |
| ENSG00000057019 | DCBLD2 | -0.389 | -0.360 | -0.339 | -0.366 | -0.319 | -0.416 |
| ENSG00000143641 | GALNT2 | -0.390 | -0.188 | -0.168 | -0.186 | -0.190 | -0.209 |
| ENSG00000100345 | MYH9 | -0.391 | -0.335 | -0.194 | -0.204 | -0.358 | -0.586 |
| ENSG00000203485 | INF2 | -0.391 | -0.198 | -0.159 | -0.196 | -0.215 | -0.221 |
| ENSG00000181467 | RAP2B | -0.393 | 0.070 | 0.062 | 0.107 | 0.044 | 0.066 |
| ENSG00000141959 | PFKL | -0.394 | -0.211 | -0.239 | -0.213 | -0.270 | -0.120 |
| ENSG00000131016 | AKAP12 | -0.394 | 0.123 | 0.106 | 0.205 | 0.162 | 0.021 |
| ENSG00000001084 | GCLC | -0.396 | -0.160 | -0.139 | -0.164 | -0.100 | -0.239 |
| ENSG00000125733 | TRIP10 | -0.396 | -0.282 | -0.204 | -0.271 | -0.335 | -0.316 |
| ENSG00000075142 | SRI | -0.397 | -0.460 | -0.254 | -0.278 | -0.284 | -1.022 |
| ENSG00000162734 | PEA15 | -0.398 | -0.322 | -0.254 | -0.549 | -0.253 | -0.231 |
| ENSG00000184292 | TACSTD2 | -0.400 | -0.129 | -0.104 | -0.115 | -0.158 | -0.140 |
| ENSG00000092621 | PHGDH | -0.403 | 0.392 | 0.090 | 0.140 | 0.080 | 1.258 |
| ENSG00000115641 | FHL2 | -0.403 | -0.036 | -0.074 | -0.096 | -0.085 | 0.109 |
| ENSG00000100139 | MICALL1 | -0.405 | -0.057 | -0.064 | -0.008 | -0.110 | -0.049 |
| ENSG00000197448 | GSTK1 | -0.406 | -0.310 | -0.259 | -0.250 | -0.317 | -0.415 |
| ENSG00000060491 | OGFR | -0.407 | -0.257 | -0.177 | -0.212 | -0.250 | -0.389 |
| ENSG00000024422 | EHD2 | -0.407 | -0.293 | -0.110 | -0.112 | -0.286 | -0.663 |
| ENSG00000087274 | ADD1 | -0.408 | 0.179 | -0.041 | -0.011 | 0.072 | 0.696 |
| ENSG00000164171 | ITGA2 | -0.411 | -0.178 | -0.119 | 0.288 | -0.189 | -0.693 |
| ENSG00000196576 | PLXNB2 | -0.419 | -0.043 | -0.186 | -0.171 | -0.185 | 0.373 |
| ENSG00000092010 | PSME1 | -0.420 | -0.321 | -0.231 | -0.286 | -0.324 | -0.442 |
| ENSG00000086598 | TMED2 | -0.420 | -0.123 | -0.217 | -0.201 | -0.049 | -0.028 |
| ENSG00000099797 | TECR | -0.421 | 0.056 | 0.034 | 0.069 | 0.084 | 0.038 |
| ENSG00000101199 | ARFGAP1 | -0.425 | -0.195 | -0.120 | -0.495 | -0.125 | -0.039 |
| ENSG00000105993 | DNAJB6 | -0.425 | -0.145 | -0.129 | -0.227 | 0.023 | -0.247 |
| ENSG00000167969 | ECI1 | -0.426 | 0.033 | 0.017 | 0.099 | 0.030 | -0.012 |
| ENSG00000175063 | UBE2C | -0.426 | -0.022 | 0.005 | -0.056 | -0.080 | 0.043 |
| ENSG00000115756 | HPCAL1 | -0.428 | -0.323 | -0.226 | -0.355 | -0.347 | -0.365 |
| ENSG00000100075 | SLC25A1 | -0.429 | 0.121 | -0.061 | -0.099 | -0.065 | 0.710 |
| ENSG00000127824 | TUBA4A | -0.435 | -0.102 | -0.080 | -0.089 | -0.136 | -0.103 |
| ENSG00000173599 | PC | -0.439 | -0.025 | -0.032 | -0.066 | -0.222 | 0.221 |
| ENSG00000170275 | CRTAP | -0.445 | -0.194 | -0.118 | -0.068 | -0.083 | -0.506 |
| ENSG00000096433 | ITPR3 | -0.446 | -0.006 | -0.041 | -0.043 | -0.088 | 0.149 |
| ENSG00000163110 | PDLIM5 | -0.446 | -0.303 | -0.282 | -0.323 | -0.155 | -0.451 |
| ENSG00000131746 | TNS4 | -0.449 | -0.149 | -0.088 | -0.032 | -0.141 | -0.336 |
| ENSG00000089159 | PXN | -0.449 | 0.154 | 0.050 | 0.095 | 0.140 | 0.329 |
| ENSG00000099812 | MISP | -0.451 | -0.142 | -0.113 | -0.129 | -0.172 | -0.151 |
| ENSG00000129116 | PALLD | -0.460 | -0.052 | -0.138 | 0.080 | -0.151 | 0.002 |
| ENSG00000064666 | CNN2 | -0.461 | -0.060 | -0.082 | 0.214 | -0.102 | -0.270 |
| ENSG00000104267 | CA2 | -0.462 | 0.326 | -0.149 | -0.177 | 0.054 | 1.575 |
| ENSG00000132589 | FLOT2 | -0.466 | -0.103 | -0.127 | -0.162 | -0.034 | -0.091 |
| ENSG00000206075 | SERPINB5 | -0.466 | -0.500 | -0.381 | -0.444 | -0.398 | -0.778 |
| ENSG00000167114 | SLC27A4 | -0.491 | 0.213 | 0.219 | 0.255 | 0.242 | 0.138 |
| ENSG00000138594 | TMOD3 | -0.497 | -0.465 | -0.357 | -0.753 | -0.294 | -0.456 |
| ENSG00000240065 | PSMB9 | -0.502 | -0.087 | -0.134 | N/A | -0.194 | 0.068 |
| ENSG00000166920 | C15orf48 | -0.502 | -0.751 | -0.605 | -0.695 | -0.822 | -0.880 |
| ENSG00000086065 | CHMP5 | -0.507 | -0.236 | -0.187 | -0.222 | -0.313 | -0.220 |
| ENSG00000126458 | RRAS | -0.512 | -0.188 | -0.238 | -0.270 | -0.308 | 0.063 |
| ENSG00000143375 | CGN | -0.514 | -0.211 | -0.195 | -0.240 | -0.294 | -0.114 |
| ENSG00000177425 | PAWR | -0.518 | 0.037 | -0.069 | -0.110 | 0.235 | 0.093 |
| ENSG00000204264 | PSMB8 | -0.528 | -0.197 | -0.185 | N/A | -0.544 | 0.138 |
| ENSG00000115221 | ITGB6 | -0.530 | -0.105 | -0.099 | -0.144 | -0.241 | 0.063 |
| ENSG00000076685 | NT5C2 | -0.541 | -0.479 | -0.385 | -0.422 | -0.653 | -0.454 |
| ENSG00000177469 | PTRF | -0.549 | -0.324 | -0.290 | -0.307 | -0.360 | -0.340 |
| ENSG00000180353 | HCLS1 | -0.554 | -0.161 | -0.163 | -0.173 | -0.237 | -0.071 |
| ENSG00000126432 | PRDX5 | -0.555 | -0.018 | -0.072 | -0.013 | -0.077 | 0.091 |
| ENSG00000160293 | VAV2 | -0.583 | 0.169 | -0.022 | -0.004 | -0.062 | 0.764 |
| ENSG00000026025 | VIM | -0.594 | -0.484 | -0.408 | -0.442 | -0.544 | -0.541 |
| ENSG00000101294 | HM13 | -0.596 | -0.451 | -0.396 | -0.287 | -0.494 | -0.628 |
| ENSG00000126777 | KTN1 | -0.598 | -0.169 | -0.206 | -0.241 | -0.032 | -0.196 |
| ENSG00000167460 | TPM4 | -0.616 | -0.460 | -0.172 | -0.596 | -0.161 | -0.911 |
| ENSG00000117394 | SLC2A1 | -0.627 | -0.054 | -0.124 | -0.379 | -0.072 | 0.358 |
| ENSG00000163220 | S100A9 | -0.629 | -0.098 | -0.058 | -0.091 | -0.137 | -0.105 |
| ENSG00000007080 | CCDC124 | -0.636 | 0.386 | 0.323 | 0.518 | 0.334 | 0.371 |
| ENSG00000173432 | SAA1 | -0.656 | -0.164 | -0.162 | -0.191 | -0.273 | -0.030 |
| ENSG00000143546 | S100A8 | -0.663 | -0.226 | -0.264 | -0.201 | -0.475 | 0.036 |
| ENSG00000167996 | FTH1 | -0.675 | -0.088 | -0.067 | 0.011 | -0.062 | -0.234 |
| ENSG00000170955 | PRKCDBP | -0.682 | -0.325 | -0.250 | -0.327 | -0.342 | -0.383 |
| ENSG00000137312 | FLOT1 | -0.696 | -0.175 | -0.171 | -0.253 | -0.067 | -0.209 |
| ENSG00000104881 | PPP1R13L | -0.697 | -0.056 | -0.007 | -0.192 | -0.166 | 0.142 |
| ENSG00000130313 | PGLS | -0.731 | -0.463 | -0.369 | -0.461 | -0.425 | -0.596 |
| ENSG00000136689 | IL1RN | -0.732 | -0.378 | -0.369 | -0.373 | -0.500 | -0.268 |
| ENSG00000120913 | PDLIM2 | -0.786 | -0.152 | -0.066 | -0.235 | -0.244 | -0.064 |
| ENSG00000101109 | STK4 | -0.870 | -0.258 | -0.213 | -0.283 | -0.325 | -0.211 |
| ENSG00000167880 | EVPL | -0.880 | 0.255 | 0.082 | 0.749 | -0.057 | 0.246 |
| ENSG00000166340 | TPP1 | -0.903 | -0.356 | -0.331 | -0.421 | -0.266 | -0.407 |
| ENSG00000118898 | PPL | -0.941 | -0.127 | -0.084 | -0.100 | -0.179 | -0.143 |
| ENSG00000148346 | LCN2 | -1.159 | -1.140 | -0.591 | -0.978 | -1.182 | -1.806 |
| ENSG00000134049 | IER3IP1 | -1.326 | -0.435 | -0.139 | -0.202 | -0.732 | -0.668 |
| ENSG00000267228 | AC012254.2 | -1.326 | 0.615 | N/A | 0.823 | 0.407 | N/A |
| ENSG00000114062 | UBE3A | -1.464 | -0.289 | -0.089 | -0.200 | -0.198 | -0.667 |
| ENSG00000143556 | S100A7 | -1.843 | -0.372 | -0.165 | -0.335 | -0.377 | -0.613 |
| ENSG00000109062 | SLC9A3R1 | -3.103 | 0.343 | 0.394 | 0.164 | 0.460 | 0.356 |
