# Supplementary material for: Combined Transcriptome and Proteome Analysis of Immortalized Human Keratinocytes Expressing Human Papillomavirus 16 (HPV16) Oncogenes Reveals Novel Key Factors and Networks in HPV-Induced Carcinogenesis
Source: mSphere. 2019 Mar 27;4(2):e00129-19. doi: 10.1128/mSphere.00129-19 (PMC6437273; doi:10.1128/mSphere.00129-19)
Supplement: TABLE S4 [file mSphere.00129-19-st004.docx]

**Table S4**

| **gene name** | **gene ID** | **average RNA** | **SILAC** | **HFK E6E7** | **HCK E6E7** | **HLF E7** | **HLF E6** |
| --- | --- | --- | --- | --- | --- | --- | --- |
| UCHL1 | ENSG00000154277 | 5.7893 | 2.5976 | 3.9857 | N/A | N/A | N/A |
| LCP1 | ENSG00000136167 | 2.3700 | 2.2093 | -0.0165 | N/A | N/A | N/A |
| CRABP2 | ENSG00000143320 | 1.8726 | 1.6246 | 1.1254 | N/A | -0.0070 | 0.8155 |
| EPPK1 | ENSG00000261150 | 1.7272 | 1.0409 | N/A | N/A | N/A | N/A |
| THBD | ENSG00000178726 | 1.6591 | 1.3267 | -0.1411 | N/A | N/A | N/A |
| HSPA2 | ENSG00000126803 | 1.2655 | 0.3864 | 2.1624 | N/A | 0.3124 | 0.4663 |
| KYNU | ENSG00000115919 | 1.0081 | 0.8030 | -0.8039 | N/A | 1.3802 | 0.5965 |
| CKB | ENSG00000166165 | 0.9241 | 1.2501 | 0.7450 | N/A | 0.1256 | 0.6165 |
| ARG2 | ENSG00000081181 | 0.9116 | 1.2493 | 0.5888 | -1.8520 | N/A | N/A |
| TP63 | ENSG00000073282 | 0.8951 | 0.5185 | 0.1665 | N/A | N/A | N/A |
| RAD50 | ENSG00000113522 | 0.8643 | 0.9504 | 0.3247 | N/A | N/A | N/A |
| INPP1 | ENSG00000151689 | 0.8632 | 0.7708 | 0.5889 | N/A | N/A | N/A |
| FAM129A | ENSG00000135842 | 0.8490 | 0.5342 | 1.3315 | N/A | 0.1328 | 0.7045 |
| HMGA1 | ENSG00000137309 | 0.8488 | 0.4314 | 0.2260 | N/A | N/A | N/A |
| HMGA2 | ENSG00000149948 | 0.8195 | 0.5281 | -0.5894 | N/A | N/A | N/A |
| PYCR1 | ENSG00000183010 | 0.8130 | 0.4439 | 0.8316 | N/A | -0.4373 | 0.0052 |
| PRMT3 | ENSG00000185238 | 0.8127 | 0.5687 | 0.4147 | N/A | N/A | N/A |
| ZC3H11A | ENSG00000058673 | 0.8101 | 0.6858 | -0.1770 | N/A | N/A | N/A |
| NSUN5 | ENSG00000130305 | 0.8021 | 0.4741 | 0.6271 | N/A | 0.1692 | 0.3965 |
| SORD | ENSG00000140263 | 0.7802 | 0.4536 | 0.3271 | N/A | N/A | N/A |
| UPP1 | ENSG00000183696 | 0.7115 | 0.5977 | -0.6908 | N/A | N/A | N/A |
| FOXK1 | ENSG00000164916 | 0.6744 | 0.4160 | -0.2822 | N/A | N/A | N/A |
| TRMT6 | ENSG00000089195 | 0.6638 | 0.4127 | 0.1306 | N/A | N/A | N/A |
| TCOF1 | ENSG00000070814 | 0.6456 | 0.5332 | 1.0698 | N/A | -0.0219 | 0.0164 |
| NSFL1C | ENSG00000088833 | 0.6447 | 0.4876 | -0.0630 | 1.5060 | N/A | N/A |
| SQSTM1 | ENSG00000161011 | 0.6327 | 0.4966 | -0.4615 | N/A | 0.1692 | 0.2901 |
| CRNKL1 | ENSG00000101343 | 0.5949 | 0.5980 | 0.6003 | N/A | N/A | N/A |
| LYAR | ENSG00000145220 | 0.5932 | 0.4508 | 0.5450 | N/A | N/A | N/A |
| SLC25A4 | ENSG00000151729 | 0.5814 | 0.5919 | 0.0706 | N/A | N/A | N/A |
| ESF1 | ENSG00000089048 | 0.5790 | 0.5905 | 0.2769 | N/A | N/A | N/A |
| RPA2 | ENSG00000117748 | 0.5684 | 0.5299 | 1.2116 | 2.0340 | 0.0455 | 0.6779 |
| PSMF1 | ENSG00000125818 | 0.5683 | 0.4478 | -0.3366 | N/A | N/A | N/A |
| ITPA | ENSG00000125877 | 0.5661 | 0.6277 | 0.2046 | 1.5590 | N/A | N/A |
| UBE2S | ENSG00000108106 | 0.5634 | 0.3937 | 1.6053 | N/A | N/A | N/A |
| SMTN | ENSG00000183963 | 0.5609 | 0.5258 | -0.1449 | N/A | N/A | N/A |
| LACTB2 | ENSG00000147592 | 0.5598 | 0.5304 | 1.1797 | N/A | N/A | N/A |
| PMVK | ENSG00000163344 | 0.5568 | 0.6539 | 0.0228 | N/A | N/A | N/A |
| TFRC | ENSG00000072274 | 0.5540 | 0.7502 | -0.0956 | N/A | 0.2082 | 0.1366 |
| AURKB | ENSG00000178999 | 0.5146 | 0.4270 | 2.4120 | N/A | 0.2900 | 0.5165 |
| PGAM5 | ENSG00000247077 | 0.5059 | 0.4021 | 0.4082 | N/A | N/A | N/A |
| EGFR | ENSG00000146648 | 0.4917 | 0.7098 | -0.5332 | N/A | N/A | N/A |
| XRN2 | ENSG00000088930 | 0.4853 | 0.4440 | 0.4485 | N/A | N/A | N/A |
| PCNA | ENSG00000132646 | 0.4783 | 0.6733 | 1.2843 | N/A | 0.1315 | 0.2008 |
| HPSE | ENSG00000173083 | 0.4662 | 0.6276 | 0.5879 | -2.3310 | N/A | N/A |
| SRXN1 | ENSG00000271303 | 0.4228 | 0.7681 | -0.3746 | N/A | N/A | N/A |
| MLKL | ENSG00000168404 | 0.4004 | 0.4739 | 0.3546 | N/A | N/A | N/A |
| TYMS | ENSG00000176890 | 0.3795 | 0.9891 | 0.9546 | 1.9570 | 0.3111 | 0.2608 |
| TRIP13 | ENSG00000071539 | 0.3780 | 0.4289 | 1.9716 | N/A | 0.0595 | 0.5077 |
| MAPRE1 | ENSG00000101367 | -0.2979 | -0.4851 | 0.2880 | N/A | N/A | N/A |
| CHMP5 | ENSG00000086065 | -0.3134 | -0.5072 | 0.1965 | N/A | N/A | N/A |
| ANXA5 | ENSG00000164111 | -0.3237 | -0.3899 | 0.1881 | 1.6910 | N/A | N/A |
| MYL12A | ENSG00000101608 | -0.3254 | -0.3861 | 0.2628 | N/A | N/A | N/A |
| CD9 | ENSG00000010278 | -0.3375 | -0.5059 | 0.0594 | N/A | 0.0446 | 0.1720 |
| SPATS2L | ENSG00000196141 | -0.3403 | -0.4377 | -0.1775 | N/A | N/A | N/A |
| PTRF | ENSG00000177469 | -0.3497 | -0.5486 | -0.0714 | 1.6740 | N/A | N/A |
| CALD1 | ENSG00000122786 | -0.3568 | -0.5411 | -0.0517 | N/A | -0.1402 | -0.3312 |
| GOPC | ENSG00000047932 | -0.3621 | -0.3816 | 0.0214 | N/A | -0.0310 | -0.2366 |
| LAP3 | ENSG00000002549 | -0.3630 | -0.4641 | 0.6283 | N/A | N/A | N/A |
| GSTK1 | ENSG00000197448 | -0.3657 | -0.4060 | -0.1912 | N/A | -0.0574 | -0.0772 |
| S100A11 | ENSG00000163191 | -0.3718 | -0.5051 | -0.8453 | N/A | N/A | N/A |
| DNAJC5 | ENSG00000101152 | -0.3756 | -0.5109 | -0.5089 | N/A | N/A | N/A |
| PSME1 | ENSG00000092010 | -0.3827 | -0.4200 | 0.3420 | N/A | N/A | N/A |
| CAV2 | ENSG00000105971 | -0.3903 | -0.4890 | 0.1348 | N/A | -0.4430 | -0.1479 |
| TRIM25 | ENSG00000121060 | -0.4078 | -0.3990 | -0.3117 | N/A | 0.2633 | 0.1898 |
| ANXA11 | ENSG00000122359 | -0.4146 | -0.5504 | -0.5983 | N/A | N/A | N/A |
| ANXA2 | ENSG00000182718 | -0.4334 | -0.4676 | -0.3430 | N/A | N/A | N/A |
| S100A10 | ENSG00000197747 | -0.4561 | -0.5592 | -0.4773 | N/A | N/A | N/A |
| CSTA | ENSG00000121552 | -0.4593 | 0.3905 | -0.8039 | N/A | N/A | N/A |
| H1F0 | ENSG00000189060 | -0.4617 | -0.5997 | -0.3700 | N/A | 0.1864 | 0.5446 |
| MOCS3 | ENSG00000124217 | -0.4651 | -0.7678 | 0.1160 | N/A | N/A | N/A |
| MYH9 | ENSG00000100345 | -0.4718 | -0.3914 | -0.2210 | N/A | N/A | N/A |
| EHD2 | ENSG00000024422 | -0.4748 | -0.4075 | -1.2289 | N/A | -0.1843 | -0.1667 |
| ITGB1 | ENSG00000150093 | -0.4776 | -0.4419 | -0.0627 | N/A | N/A | N/A |
| ANXA3 | ENSG00000138772 | -0.4814 | -0.4356 | -0.4146 | -1.9150 | -0.0883 | 0.7742 |
| SYNJ2BP | ENSG00000213463 | -0.4818 | -0.5305 | -0.3069 | N/A | N/A | N/A |
| SLC44A2 | ENSG00000129353 | -0.4848 | -0.4148 | 0.0337 | N/A | -0.1276 | 0.0056 |
| CPNE1 | ENSG00000214078 | -0.4866 | -0.7096 | -0.0338 | N/A | N/A | N/A |
| IDH1 | ENSG00000138413 | -0.4870 | -0.4493 | 0.3710 | N/A | N/A | N/A |
| OAT | ENSG00000065154 | -0.4889 | -0.4471 | 0.6214 | N/A | -0.1473 | -0.1248 |
| PLEC | ENSG00000178209 | -0.4925 | -0.4342 | -0.7573 | N/A | N/A | N/A |
| VAPB | ENSG00000124164 | -0.4972 | -0.3787 | -0.5069 | N/A | N/A | N/A |
| POFUT1 | ENSG00000101346 | -0.5028 | -0.4896 | 0.2043 | N/A | N/A | N/A |
| ADAM9 | ENSG00000168615 | -0.5045 | -0.4247 | -0.0108 | N/A | N/A | N/A |
| PGLS | ENSG00000130313 | -0.5108 | -0.7311 | -0.4230 | N/A | N/A | N/A |
| GSN | ENSG00000148180 | -0.5124 | -0.8637 | -0.8172 | -1.7780 | -0.1931 | -0.4564 |
| TRIM16 | ENSG00000221926 | -0.5150 | -0.4642 | -0.6706 | -1.6020 | N/A | N/A |
| AHNAK | ENSG00000124942 | -0.5175 | -0.4148 | -1.2272 | -1.6250 | N/A | N/A |
| C1orf116 | ENSG00000182795 | -0.5344 | -0.5511 | -1.1593 | -2.0500 | N/A | N/A |
| CNN3 | ENSG00000117519 | -0.5430 | -0.3868 | 0.5253 | N/A | -0.2225 | -0.0708 |
| MAOA | ENSG00000189221 | -0.5630 | -0.5728 | -0.5461 | N/A | N/A | N/A |
| EHBP1 | ENSG00000115504 | -0.5643 | -0.5676 | -0.6653 | N/A | N/A | N/A |
| BAIAP2 | ENSG00000175866 | -0.5745 | -0.6655 | -0.6884 | N/A | N/A | N/A |
| CTSA | ENSG00000064601 | -0.5842 | -0.4651 | -1.1875 | N/A | -0.0257 | -0.1085 |
| SERPINB5 | ENSG00000206075 | -0.5883 | -0.4664 | -0.7734 | N/A | N/A | N/A |
| ETHE1 | ENSG00000105755 | -0.5963 | -0.8798 | -0.5490 | N/A | -0.3956 | -0.3288 |
| NPC2 | ENSG00000119655 | -0.5968 | -0.4593 | 0.2972 | N/A | -0.1381 | -0.2032 |
| SVIL | ENSG00000197321 | -0.5971 | -0.5678 | -0.6693 | N/A | 0.4806 | -0.1443 |
| MARCKS | ENSG00000277443 | -0.6003 | -0.6180 | N/A | N/A | N/A | N/A |
| INPP4B | ENSG00000109452 | -0.6028 | -0.5834 | -0.6857 | N/A | N/A | N/A |
| NT5C2 | ENSG00000076685 | -0.6033 | -0.5412 | -0.4396 | N/A | N/A | N/A |
| TMOD3 | ENSG00000138594 | -0.6048 | -0.4972 | -0.2769 | N/A | N/A | N/A |
| CAV1 | ENSG00000105974 | -0.6071 | -0.5559 | 0.0223 | N/A | N/A | N/A |
| TPMT | ENSG00000137364 | -0.6081 | -0.4288 | -0.2866 | N/A | N/A | N/A |
| DTX3L | ENSG00000163840 | -0.6194 | -1.4814 | -0.2822 | N/A | -0.0602 | 0.5140 |
| TMSB4X | ENSG00000205542 | -0.6262 | -0.4620 | -0.4508 | N/A | N/A | N/A |
| MYOF | ENSG00000138119 | -0.6441 | -0.6818 | -0.4590 | N/A | N/A | N/A |
| HM13 | ENSG00000101294 | -0.6464 | -0.5957 | -0.4645 | N/A | 0.0349 | -0.1517 |
| PLSCR1 | ENSG00000188313 | -0.6519 | -0.6424 | 0.3384 | N/A | 0.1171 | 0.4897 |
| SRI | ENSG00000075142 | -0.6534 | -0.3974 | 0.3531 | N/A | N/A | N/A |
| FAM3C | ENSG00000196937 | -0.6593 | -0.5644 | -0.3937 | N/A | N/A | N/A |
| LMO7 | ENSG00000136153 | -0.6599 | -0.5677 | -1.2058 | N/A | -0.3763 | -0.1793 |
| CTSD | ENSG00000117984 | -0.6658 | -0.8564 | -1.7044 | -1.5230 | -0.0844 | -0.0414 |
| LAMA3 | ENSG00000053747 | -0.6710 | -0.6937 | -0.7734 | -1.5300 | N/A | N/A |
| YWHAB | ENSG00000166913 | -0.6834 | -0.5448 | -0.1546 | N/A | N/A | N/A |
| MYD88 | ENSG00000172936 | -0.6847 | -0.4068 | -1.0777 | N/A | N/A | N/A |
| FAHD1 | ENSG00000180185 | -0.6903 | -0.5853 | -0.2428 | N/A | N/A | N/A |
| PARP14 | ENSG00000173193 | -0.7068 | -0.8245 | 0.1400 | N/A | 0.2639 | 0.1343 |
| RAB11FIP1 | ENSG00000156675 | -0.7132 | -0.4180 | -2.1429 | -3.1170 | N/A | N/A |
| ALDH1A3 | ENSG00000184254 | -0.7365 | -0.6902 | -0.9903 | N/A | 0.0979 | 0.5697 |
| C15orf48 | ENSG00000166920 | -0.7429 | -0.5021 | -1.6109 | -3.0380 | N/A | N/A |
| CLIC4 | ENSG00000169504 | -0.7477 | -0.5332 | -0.3477 | N/A | N/A | N/A |
| RAB27B | ENSG00000041353 | -0.7528 | -0.5012 | -0.3117 | N/A | 0.5513 | 0.8532 |
| TPM4 | ENSG00000167460 | -0.7534 | -0.6159 | -0.0777 | N/A | N/A | N/A |
| SEC14L2 | ENSG00000100003 | -0.7614 | -0.5198 | -0.8849 | -3.1180 | N/A | N/A |
| IFI35 | ENSG00000068079 | -0.7643 | -0.6695 | 0.2462 | 1.6500 | 0.1876 | 0.7020 |
| C3 | ENSG00000125730 | -0.7788 | -0.6961 | -0.0561 | N/A | 3.7783 | 0.1264 |
| EIF2AK2 | ENSG00000055332 | -0.7817 | -0.4994 | -0.2101 | N/A | N/A | N/A |
| GLUL | ENSG00000135821 | -0.8081 | -0.8860 | 0.4947 | N/A | 0.0855 | 0.1716 |
| LGALS3BP | ENSG00000108679 | -0.8088 | -1.0476 | -0.0149 | N/A | 0.1554 | -0.0253 |
| CYB5R1 | ENSG00000159348 | -0.8156 | -0.7962 | -1.0581 | N/A | -0.1609 | -0.1575 |
| OAS3 | ENSG00000111331 | -0.8166 | -0.9789 | 0.1254 | N/A | 0.2850 | 0.7348 |
| DDX58 | ENSG00000107201 | -0.8455 | -0.5081 | 0.0461 | N/A | N/A | N/A |
| DPYSL2 | ENSG00000092964 | -0.8518 | -0.9962 | 0.4683 | 3.0130 | N/A | N/A |
| GPRC5A | ENSG00000013588 | -0.8529 | -0.7928 | -1.7563 | N/A | 0.3509 | 0.4834 |
| ACSL1 | ENSG00000151726 | -0.8610 | -0.9623 | -0.9584 | N/A | N/A | N/A |
| TOM1L2 | ENSG00000175662 | -0.8632 | -1.0380 | -1.0486 | N/A | -0.3007 | -0.3610 |
| HELZ2 | ENSG00000130589 | -0.8769 | -0.8953 | -0.7997 | N/A | N/A | N/A |
| ADIRF | ENSG00000148671 | -0.9219 | -0.7981 | -1.5580 | N/A | N/A | N/A |
| GBP1 | ENSG00000117228 | -1.0452 | -1.2042 | -0.5910 | N/A | -0.1401 | -0.4250 |
| SAMHD1 | ENSG00000101347 | -1.0744 | -0.9705 | 0.7938 | N/A | 0.0073 | 0.3505 |
| NNMT | ENSG00000166741 | -1.1331 | -1.0085 | -0.0564 | N/A | -0.5906 | 0.0365 |
| TGM2 | ENSG00000198959 | -1.1902 | -1.0892 | -0.6091 | N/A | 0.4743 | -0.1247 |
| AHNAK2 | ENSG00000185567 | -1.1915 | -0.8354 | -1.7984 | N/A | -0.1245 | 0.3421 |
| STAT1 | ENSG00000115415 | -1.2774 | -1.0096 | 0.0199 | N/A | 0.0197 | -0.0197 |
| OAS2 | ENSG00000111335 | -1.3255 | -1.5392 | -0.2091 | N/A | N/A | N/A |
| SAMD9 | ENSG00000205413 | -1.3261 | -1.0364 | -1.0697 | -2.7910 | N/A | N/A |
| LCN2 | ENSG00000148346 | -1.3922 | -1.1585 | -4.2578 | -9.1340 | N/A | N/A |
| ISG15 | ENSG00000187608 | -1.5873 | -1.5993 | 0.3288 | N/A | N/A | N/A |
| A2ML1 | ENSG00000166535 | -1.6341 | -1.1193 | -4.1449 | N/A | N/A | N/A |
| SLC26A2 | ENSG00000155850 | -1.6559 | -1.5444 | -0.0200 | N/A | -0.1456 | -0.2565 |
| MX1 | ENSG00000157601 | -1.7624 | -2.0746 | -0.0797 | N/A | N/A | N/A |
| FN1 | ENSG00000115414 | -1.7647 | -1.5750 | -1.3528 | N/A | N/A | N/A |
| DDX60 | ENSG00000137628 | -1.7969 | -1.1934 | 0.2431 | N/A | 0.2065 | 0.8287 |
| TAGLN | ENSG00000149591 | -1.9487 | -2.0903 | -1.6752 | N/A | N/A | N/A |
| CPPED1 | ENSG00000103381 | -3.1659 | -2.6566 | -0.5441 | N/A | -0.0869 | -0.2201 |
