# Supplementary material for: Combined Transcriptome and Proteome Analysis of Immortalized Human Keratinocytes Expressing Human Papillomavirus 16 (HPV16) Oncogenes Reveals Novel Key Factors and Networks in HPV-Induced Carcinogenesis
Source: mSphere. 2019 Mar 27;4(2):e00129-19. doi: 10.1128/mSphere.00129-19 (PMC6437273; doi:10.1128/mSphere.00129-19)
Supplement: TABLE S5 [file mSphere.00129-19-st005.docx]

**Table S5**

| **Dataset** | **all factors deregulated** | **Factors regulated by selected upstream regulators** | **%** |
| --- | --- | --- | --- |
| **All lists summary** | 3837 | 586 | 15.27 |
| **SILAC** | 290 | 90 | 31.14 |
| **BtDe** | 496 | 160 | 32.26 |
| **HsStDe** | 2155 | 355 | 16.47 |
| **SmDe** | 3063 | 430 | 14.04 |
| **SmSu** | 2212 | 375 | 16.95 |
