# Supplementary material for: Combined Transcriptome and Proteome Analysis of Immortalized Human Keratinocytes Expressing Human Papillomavirus 16 (HPV16) Oncogenes Reveals Novel Key Factors and Networks in HPV-Induced Carcinogenesis
Source: mSphere. 2019 Mar 27;4(2):e00129-19. doi: 10.1128/mSphere.00129-19 (PMC6437273; doi:10.1128/mSphere.00129-19)
Supplement: TABLE S6 [file mSphere.00129-19-st006.docx]

**Table S6**

| **Upstream regulators** | **silac289** | **BtDe** | **HsStDe** | **SmDe** | **SmSu** | **HFK E6E7** | **HCK E6E7** | **HLF E6** | **HLF E7** |
| --- | --- | --- | --- | --- | --- | --- | --- | --- | --- |
| RABL6 | 1.3416 | 1.7465 | 2.5557 | 2.5776 | 2.2014 | Activated | Activated | Activated | N/A |
| CCND1 | N/A | 0.4781 | 0.0739 | N/A | 1.5181 | Activated | N/A | Activated | N/A |
| CDKN1A | N/A | -1.0661 | -1.3965 | -1.8687 | -1.3871 | Inhibited | Inhibited | Inhibited | Inhibited |
| EHF | -0.7071 | -1.8974 | -1.5000 | -0.9428 | -1.2127 | Inhibited | Inhibited | Activated | No change |
| TNF | -1.2938 | -0.7793 | -0.8605 | -1.3048 | -2.7701 | N/A | Inhibited | No change | Activated |
| TGFB1 | -2.9382 | -2.4710 | -1.9340 | -3.4647 | -3.4552 | Inhibited | Inhibited | Inhibited | Activated |
| CREB1 | N/A | 0.6402 | N/A | N/A | N/A | N/A | N/A | Activated | Activated |
